# CloMet: A Novel Open-Source and Modular Software Platform That Connects Established Metabolomics Repositories and Data Analysis Resources

**DOI:** 10.1021/acs.jproteome.2c00602

**Published:** 2023-07-10

**Authors:** Jordi Rodeiro, Ester Vidaña-Vila, Joan Navarro, Roger Mallol

**Affiliations:** † Human Environment Research, La Salle - Universitat Ramon Llull, 08022 Barcelona, Spain; ‡ Research Group on Smart Society, La Salle - Universitat Ramon Llull, 08022 Barcelona, Spain

**Keywords:** metabolomics, NMR, databases, data sharing, data mining, data analysis

## Abstract

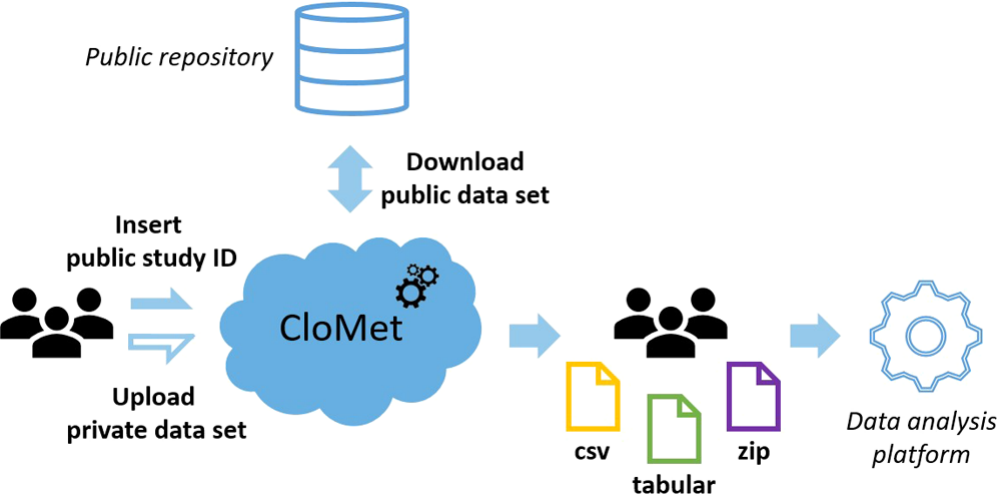

The field of metabolomics has witnessed the development of hundreds of computational tools, but only a few have become cornerstones of this field. While MetaboLights and Metabolomics Workbench are two well-established data repositories for metabolomics data sets, Workflows4Metabolomics and MetaboAnalyst are two well-established web-based data analysis platforms for metabolomics. Yet, the raw data stored in the aforementioned repositories lack standardization in terms of the file system format used to store the associated acquisition files. Consequently, it is not straightforward to reuse available data sets as input data in the above-mentioned data analysis resources, especially for non-expert users. This paper presents CloMet, a novel open-source modular software platform that contributes to standardization, reusability, and reproducibility in the metabolomics field. CloMet, which is available through a Docker file, converts raw and NMR-based metabolomics data from MetaboLights and Metabolomics Workbench to a file format that can be used directly either in MetaboAnalyst or in Workflows4Metabolomics. We validated both CloMet and the output data using data sets from these repositories. Overall, CloMet fills the gap between well-established data repositories and web-based statistical platforms and contributes to the consolidation of a data-driven perspective of the metabolomics field by leveraging and connecting existing data and resources.

## 1 Introduction

Metabolomics is the science that studies metabolites.^1^ Metabolites are small molecules that participate in general metabolic reactions and that are required for the maintenance, growth, and normal function of a cell.^2^ The identification and quantification of such metabolites provide unique insights into the metabolic processes that are taking place in the cellular environment.^2,3^ This relatively new scientific field^4^ is already finding applications in a wide variety of research fields including disease diagnosis, drug toxicity studies, and functional genomics.^5^ Overall, metabolomics studies allow us to (1) get closer to the phenotype, (2) decipher organismal behavior and functions, (3) define health and disease statuses, and (4) provide translatable evidence for various fields.^4^

**Figure 1:**
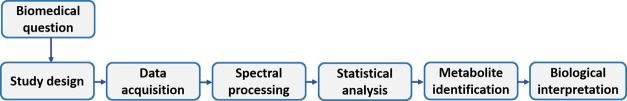
Untargeted metabolomics workflow.

A metabolomics study is composed of several steps, which combined form the so-called metabolomics workflow (Figure 1). This metabolomics workflow starts with the formulation of a biomedical question, which is the hypothesis that the researcher raises. Then, data are acquired through metabolomic experiments. Nuclear magnetic resonance (NMR) spectroscopy, gas chromatography–mass spectrometry (GC–MS), and liquid chromatography–mass spectrometry (LC–MS) are the most frequently employed bioanalytical methods.^5−7^ The acquired spectral data are (pre-)processed and statistically analyzed, steps that are followed by the biological interpretation of the results obtained. Comprehensive reviews about software tools, databases, and resources for metabolomics can be found elsewhere.^4,8^

Because we are interested in mining and analyzing spectral metabolomics data, the metabolomics workflow shown in Figure 1 corresponds to the untargeted metabolomics approach, where metabolites are identified *a posteriori*. Of the several challenges that the metabolomics field needs to tackle before reaching maturity, the lack of standards for metabolomics data and software tools is of paramount importance.^9^ As every manufacturer develops new features, uses different digitization technologies, and different acquisition methods, the complexity of data formats increases^10^ and valuable data sets could be lost to the community unless an appropriate controlled-access infrastructure is implemented.^11^ In parallel, the lack of standards regarding metabolomics tools limits compatibility^12^ and it often results in tools not getting adopted by the community.^13^ In this context, a joint effort is needed to implement FAIR (which stands for Findability, Accessibility, Interoperability, and Reusability) practices^13^ that unify current analytical data,^10^ and the same principles should apply not only for metabolomics data but also for metabolomics tools.

**Figure 2:**
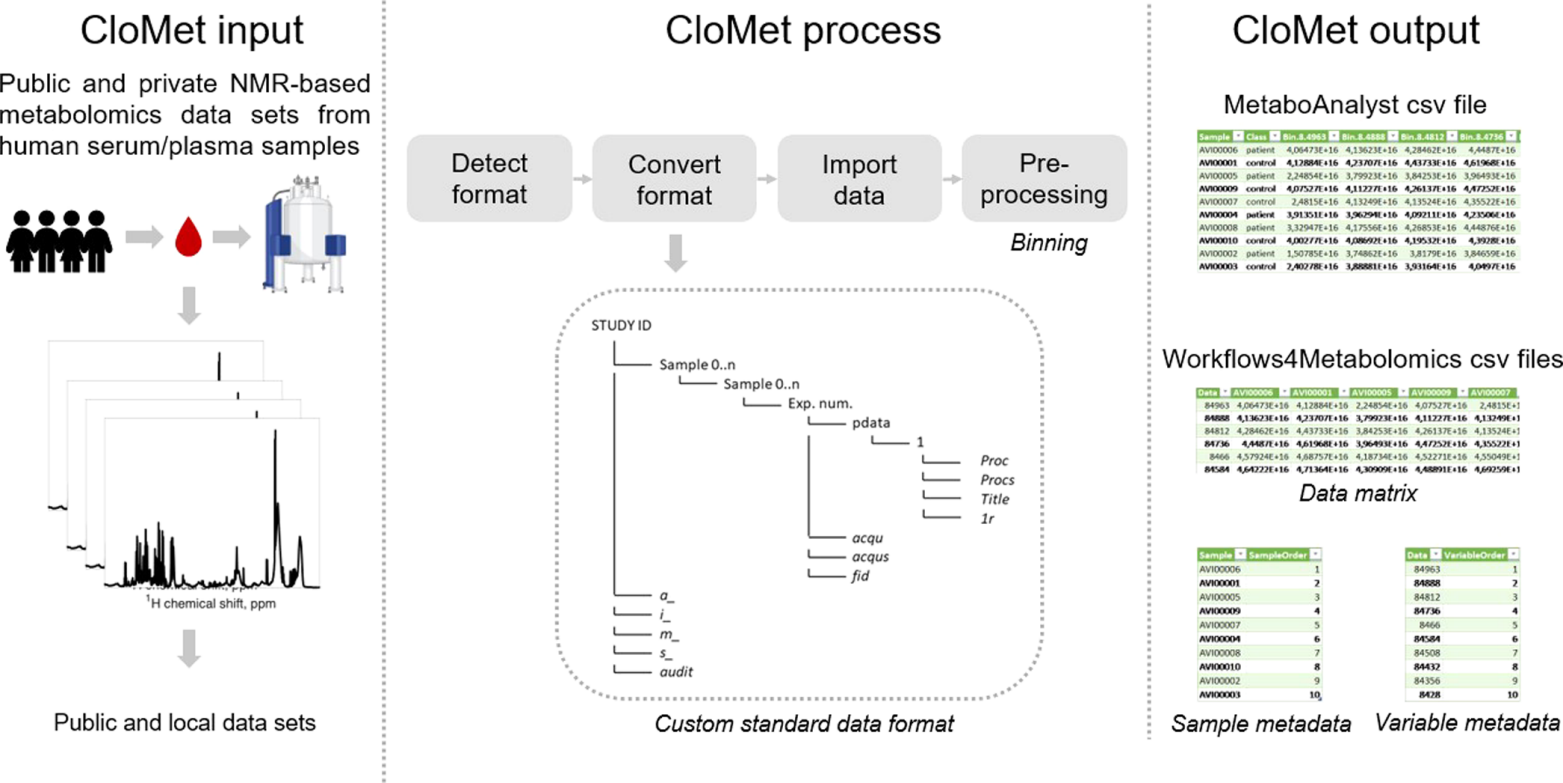
General scheme of CloMet inputs, processes, and outputs.

In summary, the development of a data standardization framework for metabolomics can contribute to overcome the aforementioned limitations and to consolidate the metabolomics field by, for example, helping researchers (1) to reuse data uploaded to online repositories by other researchers and (2) to foster the adoption of already available platforms for data deposition and analysis. To this end, we developed CloMet, a novel open-source and modular software platform that contributes to standardization, reusability, and reproducibility in the metabolomics field. In this first version, CloMet connects established metabolomics data repositories and data analysis platforms by standardizing available public data sets that have been obtained by NMR spectroscopy, with a focus on untargeted metabolomics (Figure 2).

## 2 CloMet Description

### 2.1 Input Requirements

#### 2.1.1 Analysis of Established Data Repositories

In order to design and develop CloMet, we first focused on defining its input data. Since CloMet obtains data from public online repositories, we had to decide which input repositories it could accept. Indeed, this decision directly affects the file formats accepted and used by our platform. The largest and more used public online repositories are MetaboLights and Metabolomics Workbench.^3,14^ That is the main reason why we decided to work with those as input repositories. Both online repositories are designed in a different way, store data differently, and include raw data with variable formats (see Supporting Table S1). Therefore, we studied them separately to design CloMet in such a way it can adapt to the particularities of both repositories. Also, since these two repositories store hundreds of metabolomic studies and CloMet is a tool specially designed for untargeted NMR analysis, we decided to limit the analysis to studies that (1) involved human blood serum or plasma samples, (2) analyzed them using the NMR technique, and (3) had the raw data uploaded to the repository. This does not mean that CloMet would not work on other studies, for example, those performed on other species such as mice, but that only the raw data formats belonging to this group were categorized.

#### 2.1.2 Analysis of MetaboLights Repository

**Figure 3:**
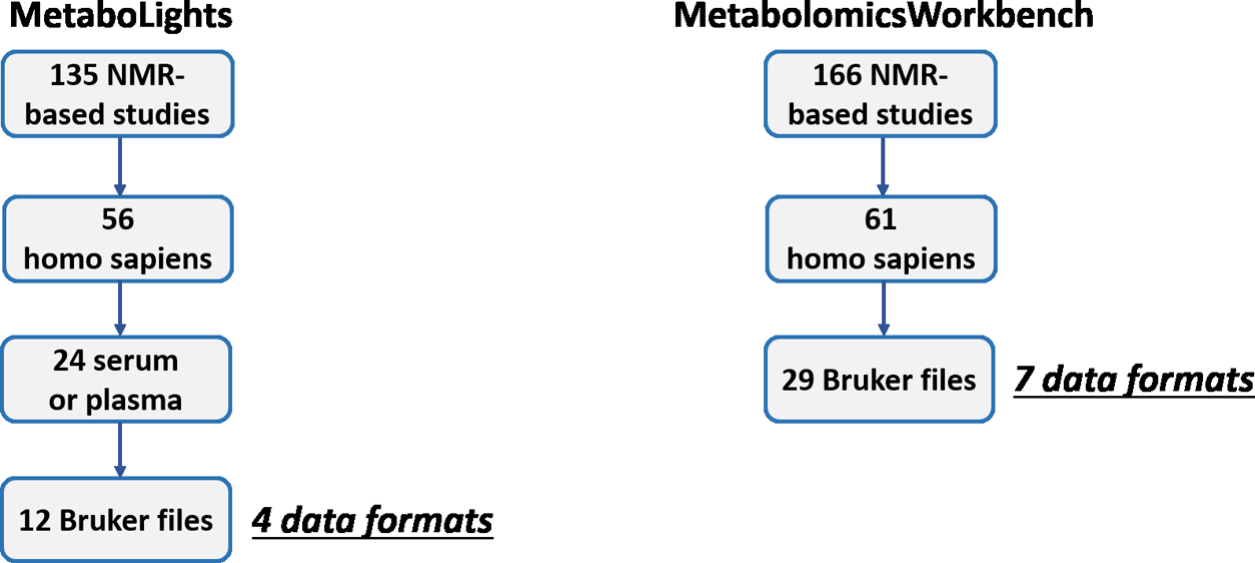
Analysis of established metabolomics data repositories.

MetaboLights stores a total of 887 studies, 135 of which used the NMR data acquisition technique (see left column in Figure 3). Of these 135, 56 corresponded to studies that involved the analysis of human samples: 7 analyzed blood plasma and 17 analyzed blood serum. Together, these 24 studies that analyzed either blood plasma or serum samples contain more than 11,000 samples. Importantly, all the selected studies (24) were acquired using a Bruker spectrometer and explained the data acquisition technique (NMR experiment) used. However, different versions of Bruker instruments were found across studies, as well as different experiment types. Therefore, there were several data format systems that had to be analyzed and standardized. Of note, 4 data sets did not present data regarding the experiment type used and were excluded. Of the 24 studies that fit the search parameters, 7 of them were discarded due to not having enough data available on MetaboLights (data missing completely or some important files missing). Some of them contained enough information to be able to analyze the file system format and were considered to improve the system. However, CloMet could not work with them, and they were discarded for the concrete analysis and tool testing. The remaining 17 studies had different file formats: .fid, .mzXML, .CNX, .nmrML, and Bruker directory (with different folder system formats). Of those, it was decided to focus on the Bruker ones, since they are the majority, and special software is needed to open the other formats. 9 studies remained after several rounds of analysis. From these 9 studies, we distinguished 4 different formats, which had to be detected and converted to a standard in order to acquire the data that they contained.

#### 2.1.3 Analysis of Metabolomics Workbench Repository

Metabolomics Workbench contains 1592 studies (see right column in Figure 3). Of these, 166 were performed using the NMR technique. 61 of the NMR studies were performed in homo sapiens, and 41 contain raw data. Of these, 9 contain blood plasma analysis, and 7 studied blood serum samples. It was decided to include all 41 studies for the Metabolomics Workbench format analysis in order to better understand the existing formats. Contrary to MetaboLights, not only Bruker machine performed experiments were found, but Agilent/Varian performed experiments are also part of the selected sample: 33 Bruker experiments and 8 Agilent/Varian. Despite most of the experiments type are NOESY, other alternatives are also present. One study was discarded due to not having any information except for the study data format. Metabolomics Workbench contains 41 studies that fit the extended search parameters. Of the 41 studies analyzed, 5 of them were discarded due to not having enough data available on Metabolomics Workbench (data missing completely or some important files missing). Some of them contained enough information to be able to analyze the file system format and were considered to improve the system. However, CloMet could not work with them, and they were discarded for concrete analysis and tool testing. The remaining 36 studies had two different file formats: .fid and Bruker (with different folder system formats). As in the previous case, it was decided to focus on the Bruker ones since they are the majority, and special software is needed to open the other format. 29 studies remained after several rounds of analysis. In this case, we distinguished 7 different formats, which were also detected and converted.

#### 2.1.4 Definition of a Custom Standard Format

**Figure 4:**
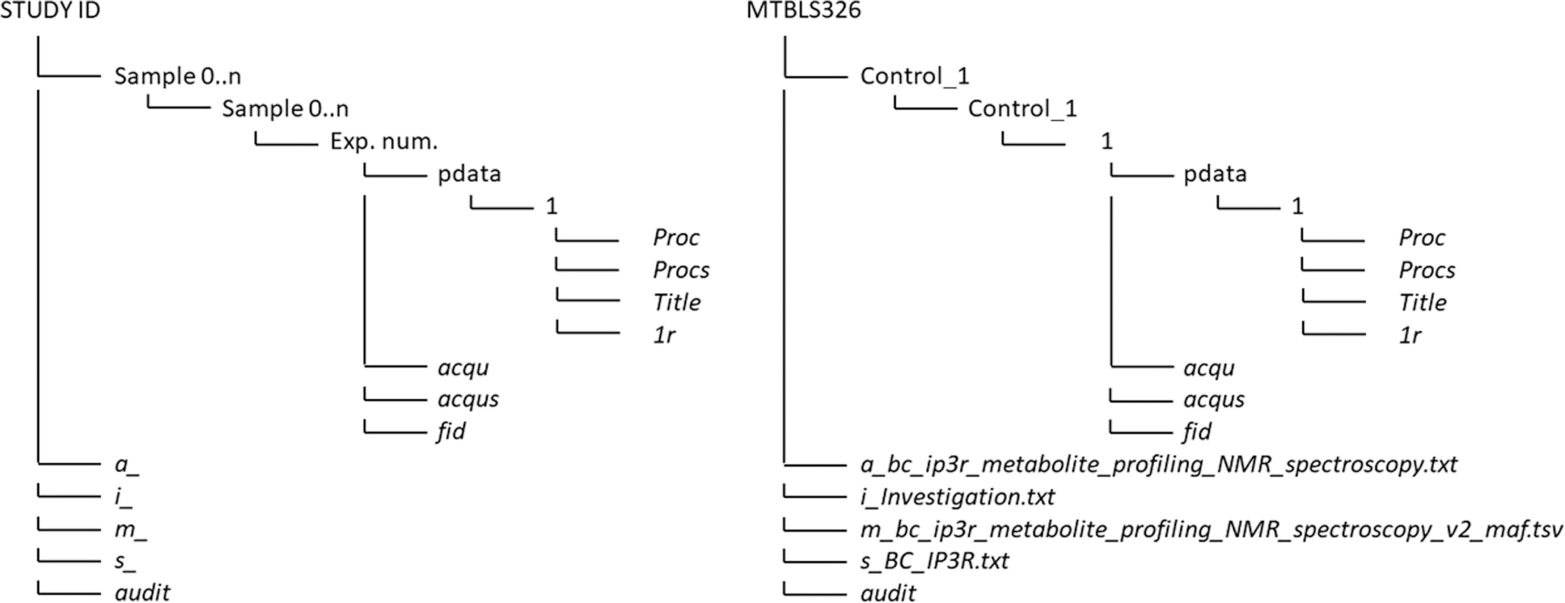
Custom standard data format. The left diagram shows the general structure of any study. The right diagram shows an example of a processed study with id MTBL326.

With all the information available regarding the input formats, we were able to define our custom standard format (see Figure 4). CloMet has the capability of detecting the input data format and transforming it to the custom format that is created by our platform. This standard simplifies the data import, spectral processing, and output format conversion steps. All the files and data belonging to a particular study are placed inside a directory with the study ID. On the first level, there are the basic files that all metabolomics studies contain. These are labeled as a_, i_, m_, s_ and audit in the left diagram in Figure 4. This is because they can have different names (as seen on the right diagram of the same figure), but they always start with the stated character (a, i, m, s) and an underscore (_). On the same level, there are also the directories that contain each one of the samples. Each of these directories has a subdirectory with the same name. Inside it, there is another directory (or directories) with the experiment number (or numbers). Inside each experiment directory, there are the acqu, acqus, and fid files and the pdata directory. This directory contains a subdirectory that stores other important files of the metabolomics analysis. As it can be seen, every study will have different file and directory names. CloMet will not look at the names but only the file structure. Something to consider is that CloMet also validates the study. For example, any study that does not contain the 1*r* file (i.e., the file that contains the real part of the data for a 1D spectrum) in any of the samples will be considered invalid since it is one of the key files when trying to import the raw data. On the other hand, if studies contain more data, these additional data will be ignored since they are not necessary for the analysis performed by CloMet.

### 2.2 Output Requirements

#### 2.2.1 Analysis of Established Data Analysis Platforms

A second analysis involved the selection of the output tools accepted by CloMet. This directly affects the output formats created by the platform. For our platform, we also selected the two most established and supported platforms, namely, Metaboanalyst and Workflow4Metabolomics^5,15−17^ (in particular, the Statistical Analysis section), and considered the different input data formats they require. Indeed, MetaboAnalyst and Workflow4Metabolomics require different input characteristics that have to be considered. To deal with the different input formats required by the aforementioned data analysis platforms, CloMet creates an intermediate csv file in which each row is the data belonging to a sample and each column is a variable or a point from the NMR spectra. This format will be modified to fit the output format of both MetaboAnalyst and Workflow4Metabolomics.

#### 2.2.2 Analysis of MetaboAnalyst Platform

**Figure 5:**
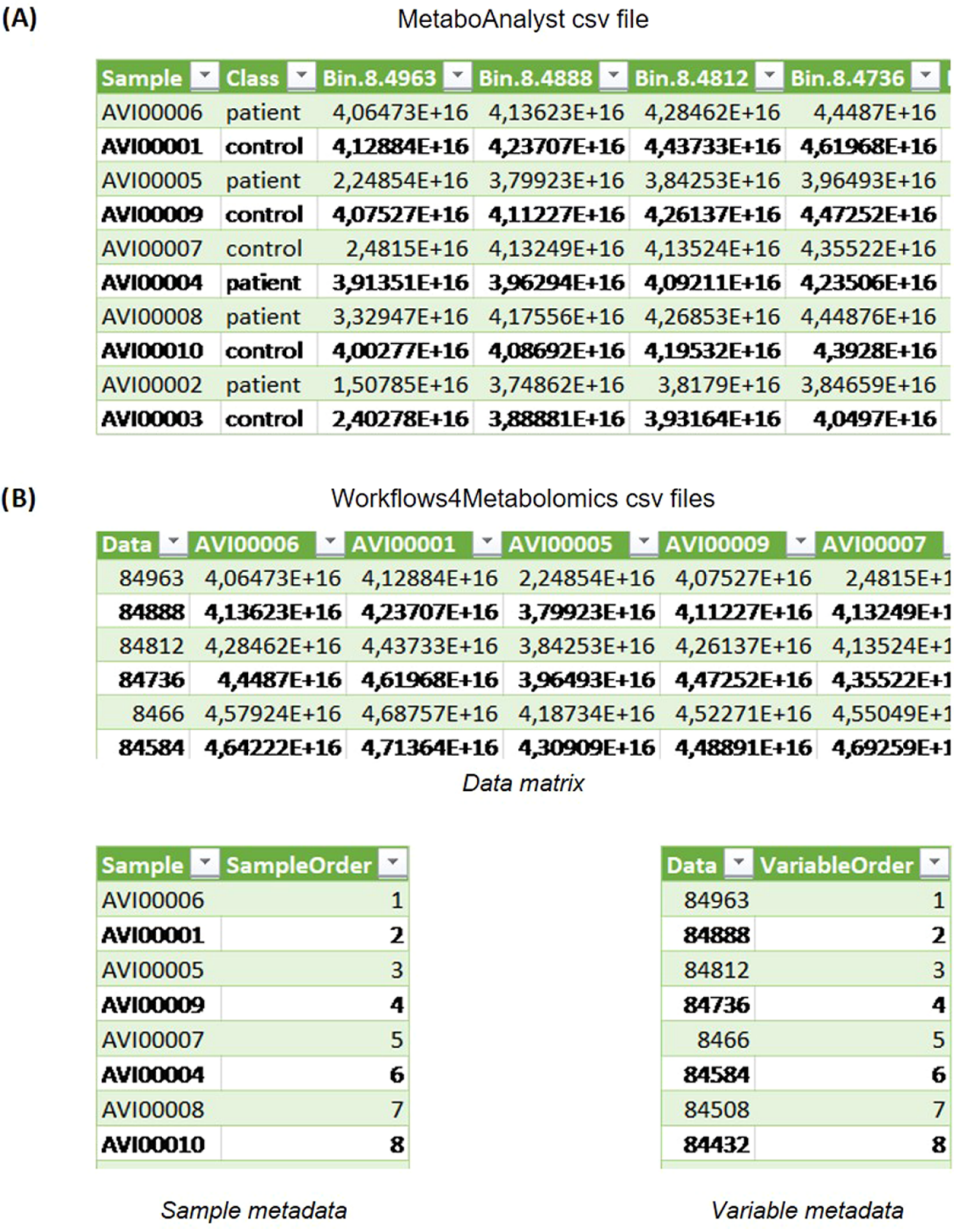
(A) MetaboAnalyst and (B) Workflow4Metabolomics csv formats.

MetaboAnalyst contains a wide range of features. For the ones that researchers are likely to use after obtaining the output from CloMet, MetaboAnalyst requires a csv file with a particular format. The format is presented in Figure 5A, taking as an example the MetaboLights study MTBLS46. Each row is the data belonging to a sample. The first column contains the study sample names. These names are set by the researcher that performs the NMR test initially and do not need to have any particular format. The second column contains the class of the sample: patient or control. In the case of the MetaboLights study MTBLS46, this means people with cardiovascular problems (patient) and people without cardiovascular problems (control). The rest of the columns belong to points in the NMR spectra. The first row of each column must have the spectral point, with the format presented in the image: Bin.SpectraPoint (ex. Bin.8.4963). To note, sample classes (e.g., case/control) must be added manually to the final csv file obtained through CloMet. For a given data set, if labels are available in either MetaboLights or Metabolomics Workbench, this step is straightforward.

#### 2.2.3 Analysis of Workflows4Metabolomics Platform

Workflow4Metabolomics also contains a wide range of options regarding both NMR and MS techniques. For CloMet, the required inputs for the following functions will be considered: NMR_Read, NMR_Preprocessing, NMR_Spectra_Alignment, NMR_Bucketing, and NMR_Multivariate. For these, Workflow4Metabolomics requires either a data matrix file, a sample data file, and a variable metadata file with particular formatting, presented in a .TABULAR file or a compressed file with the study data. For the features that require a compressed zip file with the study samples, the custom standard format created can be reformatted in order to fit the requirements. For the features that require the three .TABULAR files, the input looks like the one shown in Figure 5B. The data matrix input is very similar to the MetaboAnalyst input, with the data transposed. In this case, each column is an NMR sample, while each row is a particular spectral feature. The spectral features presented in the first column do not require any particular format. The sample metadata input contains a table with sample names and the order that they are presented in the data matrix file. In this case, the sample AVI00006 appears in the first column of the previously presented data matrix. Therefore, it will have a 1 in the sample metadata table. The variable metadata input contains a table with variable names and the order that they are presented in the data matrix file. Following the previous example, the data point 84963 appears in the first row of the data matrix file. Therefore, it will have value 1 in the VariableOrder column of the variable metadata file.

### 2.3 CloMet Processes

#### 2.3.1 Data Set Loading and Format Conversion

In order to facilitate code comprehension, reusability, and extensibility, it was decided to make CloMet object-oriented and to develop a class diagram (available on GitHub) that takes into account these design considerations. Briefly, *LoadDataset* is the first module to be executed. This module searches for the study ID on MetaboLights or Metabolomics Workbench and performs web scrapping techniques to download the data set. Of note, any other data set acquisition alternative can be added in the future. After storing the data set, the format is detected through the *DetectFormat* module. Each detected format needs different conversion steps (adding directories, removing directories, changing names···). In terms of extensibility, other formats could be added and converted here by just implementing the appropriate transformation.

#### 2.3.2 Data Import, Reduction, and Final Transformation

Once the file system is standardized, the system is able to read certain parameters from the study. The *Parameters* module enables the system to (1) check that all necessary files exist and to (2) read the pulprog belonging to every experiment type. The user can then select the desired experiment type on the graphical user interface (see Section 2.4). Besides, *ImportData* selects the necessary files from every sample to create the data matrix. Each row of this matrix is a sample obtained (a person) in the study, and every column is a different variable from the spectra. This matrix is cleaned and aligned so that the next analysis step is performed appropriately. Once the data have been imported, any pre-processing and data reduction technique can be applied. In this version, we have implemented the data binning technique. Of note, we just provide a binning version of the raw data set to allow our users to have a more manageable data set in case they just want to perform some initial exploratory analysis. The binned data set we provided relies on the spectral data contained in the 1r file, and we apply binning so that the final binned data set contains exactly 1000 data columns. Researchers aiming to further develop CloMet will be able to add any other technique and combine the different available techniques to compare the results. Also, the researcher can always download the raw data and process them again through Workflows4Metabolomics. The next step is to prepare the data for one of the two possible output formats. Depending on the tool used, a different number of files will be created. For MetaboAnalyst, the data matrix will be converted to a csv file with the required format. For Worflow4Metabolomics, three .TABULAR files will be created containing different shapes and information. These files will be presented on the web application so that the user can download them.

### 2.4 CloMet Components

#### 2.4.1 Web Application

**Figure 6:**
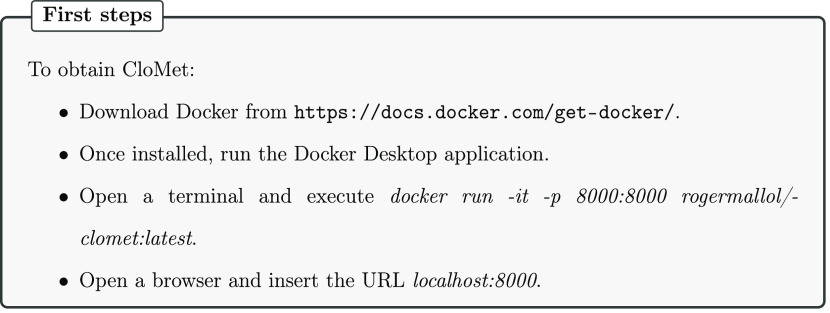
Step-by-step guide to install Docker for the first time and run CloMet.

As stated before, CloMet is designed and implemented as an open-source, modular software platform that contributes to standardization, reusability, and reproducibility in the metabolomics field (Figure 6). Importantly, being modular and open-source means that users will be able to modify CloMet. Indeed, tool extensibility is a must with the way metabolomics analysis is progressing.^18^ In this regard, it was decided to use Python as the main programming language and Django as the web framework. HTML, CSS, and JavaScript were also used in order to style the website and its components.

#### 2.4.2 Graphical User Interface (GUI)

**Figure 7:**
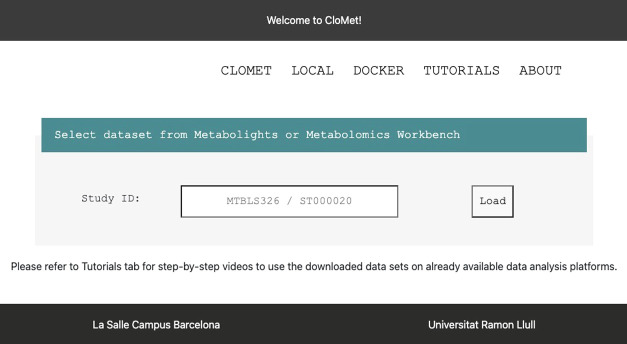
CloMet graphical user interface.

The CloMet page is the main page of the web application (Figure 7). Here, researchers will be able to download studies from online public repositories such as MetaboLights and Metabolomics Workbench. To start the process, the researcher must introduce the study ID on the blank space and press the button *Load*. The study id starts with MTBLS or ST depending on the repository selected. Then, the study is downloaded, its format is detected, and it is converted to the standard format. The user can then perform the next step. To choose the experiment type, options are presented as a dropdown menu and the user can select one of them and press *ImportData* (not shown). The server will get the data files, create the data matrix, format the matrix to adapt to the different output files, and will create the output files with the appropriate format. These files are either presented with the final extension (when just 1 file is presented) or are presented as a zip file containing all the required documents (when more than 1 file is needed). In the end, some additional data are provided to the user. These include the raw data study as found on the online repository, the customized study, and the one that can be used as an input for some functionalities in Workflow4Metabolomics. At any point, the user can restart the process by selecting a different study or choosing a different experiment type. Depending on the study size, the download may take some time (minutes). The Local page is the page where researchers will be able to standardize their studies and obtain the information in a file format that can be used by the data analysis applications. To start the process in this page, the researcher must press Choose File and upload the desired study as a zip file. By pressing Load, the study is uploaded. The rest of the process is the same as in the CloMet page.

#### 2.4.3 CloMet Container

Finally, creating a software container may also be useful since they are easy to install and allow for a combination of tools into analysis pipelines.^19^ Therefore, we made CloMet available through a Docker file. To obtain CloMet, Docker must be first downloaded from the official page (https://docs.docker.com/get-docker/). Then, a terminal must be opened, and the command “docker run −rm -it -p 8000:8000 rogermallol/clomet:latest” must be executed to download the image and execute it. Once the previous step finishes, a regular browser has to be opened and “localhost:8000” has to be inserted as a URL and the CloMet page will appear.

## 3 Validation

### 3.1 Data Conversion

**Figure 8:**
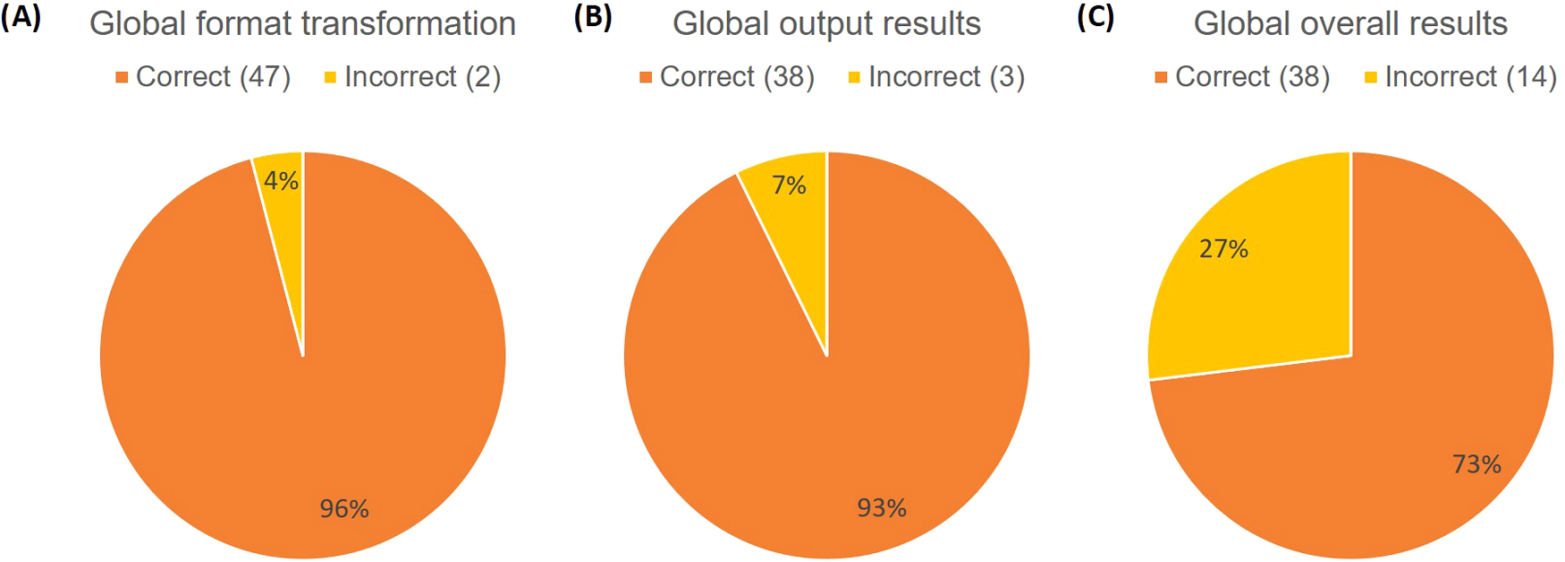
Validation results.

All the data sets that fit the selection criteria previously mentioned, both for MetaboLights and for Metabolomics Workbench, were used in order to see if the format could be transformed, and if the output could be created. For the transformation analysis, all the studies that had enough information in order to assign a format were selected. That means that studies containing data formats that CloMet cannot process were included in this testing. Researchers may just need to standardize their data, so this functionality is potentially useful. Out of the 49 data sets selected, 47 of them could be transformed (95.9%) (Figure 8A). Just considering the studies that were considered correct, 39 out of 41 studies were transformed appropriately (95.1%). When it comes to analyzing the whole process, all the accepted data sets were tested in order to obtain these results (Figure 8B). Out of the 41 accepted data sets, 38 of them obtained the correct output (92.7%). Results can also be presented for all the data sets analyzed that contained enough information. That is, the ones that contain the samples in all the possible formats, only the Bruker one (Figure 8C). The same number of data sets (38) could obtain the desired output out of the 52 studies containing data (73.1%). From these graphs, it can be said that very good results regarding obtaining the output files to be used in MetaboAnalyst or Workflow4Metabolomics were obtained.

### 3.2 Data Analysis

We downloaded four different data sets available in MetaboLights and analyzed them either in Workflows4Metabolomics or MetaboAnalyst. The four data sets were acquired using different biofluids, thus showing the wide applicability of our tool. Data sets with ids MTBLS326 and MTBLS563 corresponded to studies that used human blood serum as a specimen. Data set with id MTBLS869 was obtained through the analysis of human brain biopsies. Finally, the data set with id MTBLS431 was obtained through the analysis of *Pseudomonas aeruginosa*. Data sets MTBLS326, MTBLS431, and MTBLS869 were processed using Workflows4Metabolomics (see Supporting Figures S1–S3), while data set MTBLS563 was analyzed through MetaboAnalyst (see Supporting Figures S4 and S5). For the latter, we were able to obtain excellent discrimination between bacterial and control samples (AUC = 0.82) and viral and control samples (AUC = 0.84), in line with previously published results.

## 4 Discussion

We have presented CloMet, a novel tool to mine publicly available metabolomics data. The rationale behind CloMet is to provide an easy-to-use tool to mine existing raw and NMR-based metabolomics data by converting the original format (file system) into a format suitable for existing data analysis platforms such as MetaboAnalyst and Workflows4Metabolomics. Importantly, our aim is not to compete with these already existing tools but to fill an important gap within the metabolomics workflow: data accessibility. Indeed, as we have shown in our Supporting documentation, many public data sets are not reusable because of the format they have been shared. Through CloMet, our users will be able to easily plug their output data into one of the existing data analysis tools, being MetaboAnalyst and Workflows4Metabolomics the most used and backed-up ones. In this sense, the user will be able to perform data processing steps in those platforms, not in CloMet. Besides, CloMet allows the user to translate their own private data. This will allow, for example, NMR-based metabolomics facilities will be able to use CloMet to convert their stored data sets into a suitable format for existing data analysis platforms. This option can be found in the “LOCAL” tab of our interface. Therefore, our platform is not limited to data sets stored in public repositories but can also be used locally by any researcher holding NMR-based metabolomics data privately.

The open nature of the software (CloMet is available through GitHub) intrinsically relies on the developers’ community to extend and maintain it as it is already being done with other tools in the field (see https://github.com/topics/metabolomics). Altogether, CloMet has been designed in such a modular way meeting the Object-Oriented Programming standards to facilitate the extension of its capabilities. To further ease this task, we have released the class diagram (also available through GitHub) of the software, so developers can go directly to those modules that they want to improve/extend. While it is true that the openness nature of CloMet will allow other developers to complement CloMet with data processing algorithms, this is out of our scope. Our approach is that by just inserting a study id our users can get a ready-to-use data set with the minimal interaction possible. Besides, we envision future collaborations with these existing platforms and major software and data providers to integrate these complementary services.

As far as CloMet FAIRness is concerned, CloMet inherits the FAIR principles of the data coming from Metabolights and Metabolomics Workbench. These two repositories already promote FAIR principles.^20,21^ Thus, CloMet does not add any new indexing scheme nor modifies any file format, but it just adopts the data properties of its sources. Our custom data format is an internal standard that harmonizes the different formats found in public repositories. This custom format is also based on directories (1) to maintain the raw data as it is accessed and (2) to provide an actual format that is required to run available tools such as NMR_Read, NMR_Preprocessing, or NMR_Bucketing found in Workflows4Metabolomics. Overall, this standard simplifies the standardizing, data import, and output format conversion steps. We encourage NMR experimentalists to use a common file system and folder organization when generating a new data set (i.e., running an NMR study). This will further facilitate the uptake of shared data sets by other researchers. On the other hand, it is worth mentioning that the data stored in public repositories can either have associated information about classes or not. If a researcher also provided classes when uploading the metabolomics data, getting this information and incorporating it into the final data set is straightforward. Unfortunately, if information about classes is not available along with the metabolomics data in public repositories, re-analysis oriented to classification problems won’t be feasible. We thus also encourage the metabolomics community to also share more complete metadata to make the uploaded data sets really reusable and the associated studies replicated.

## 5 Conclusions

From our own experience, accessing available metabolomics data sets from public repositories and exploiting them through MetaboAnalyst or Workflows4Metabolomics is not a straightforward endeavor. This is specially critical for non-expert users, who may lack the computational skills needed to overcome data mining bottlenecks as a consequence of the lack of standardization of available data and tools. Here, we presented CLoMet, a novel computational tool for NMR-based untargeted metabolomics aimed at easing the connection between data repositories and data analysis platforms. Importantly, we have designed and implemented CloMet so it can be easily used by any interested researcher and further developed by those involved or interested in the development of computational tools for metabolomics. Overall, we aim to contribute to the consolidation of the metabolomics field by providing an easy approach to mining existing data and a standard data format that can be used in well-established data analysis platforms.

## Data Availability

CloMet code, docker file, and documentation are at https://github.com/rmallol/clomet.

## Supporting Information Available

MTBLS326-[NMR_Preprocessing_graph]: Individual spectra (*n* = 46) from MetaboLights study MTBLS326 after being read and preprocessed through Workflows4Metabolomics (Figure S1); MTBLS431-[NMR_Preprocessing_graph]: Individual spectra (*n* = 10) from MetaboLights study MTBLS431 after being read and preprocessed through Workflows4Metabolomics (Figure S2); MTBLS869-[NMR_Preprocessing_graph]: Individual spectra (*n* = 71) from MetaboLights study MTBLS869 after being read and preprocessed through Workflows4Metabolomics (Figure S3); ROC curve analysis: ROC curve analysis from MetaboLights study MTBLS563 to discriminate between control and bacterial samples and between control and viral samples (Figures S4 and S5) (PDF)
Analysis of established data repositories: Comprehensive analysis of NMR-based data sets (and their formats) acquired from human serum/plasma samples and available through MetaboLights and Metabolomics Workbench (Table S1) (XLSX)
