# Supplementary material for: CloMet: A Novel Open-Source and Modular Software Platform That Connects Established Metabolomics Repositories and Data Analysis Resources
Source: J Proteome Res. 2023 Jul 10;22(8):2540–7. doi: 10.1021/acs.jproteome.2c00602 (PMC10857572; doi:10.1021/acs.jproteome.2c00602)
Supplement: Supplementary file 1 — pr2c00602_si_001.pdf [file pr2c00602_si_001.pdf]

Supporting Information

CloMet: A novel open-source and modular software platform that connects established metabolomics repositories and data analysis resources

Jordi Rodeiro[1], Ester Vidaña-Vila[1], Joan Navarro[2,], and Roger Mallol[1]*

[1]Human Environment Research, La Salle - Universitat Ramon Llull, 08022, Barcelona, Spain

[2]Research Group on Smart Society, La Salle - Universitat Ramon Llull, 08022, Barcelona, Spain

Table S1. Analysis of established data repositories (XLSX): Comprehensive analysis of NMR-based data sets (and their formats) acquired from human serum/plasma samples and available through MetaboLights and Metabolomics Workbench.

Figure S1. Individual spectra (n = 46) from MetaboLights study MTBLS326 after being read and preprocessed through Workflows4Metabolomics.

Figure S2. Individual spectra (n = 10) from MetaboLights study MTBLS431 after being read and preprocessed through Workflows4Metabolomics.

Figure S3. Individual spectra (n = 71) from MetaboLights study MTBLS869 after being read and preprocessed through Workflows4Metabolomics.

Figures S4-5. ROC curve analysis from MetaboLights study MTBLS563 to discriminate between control and bacterial samples, and between control and viral samples.

MetaboLights study MTBLS326

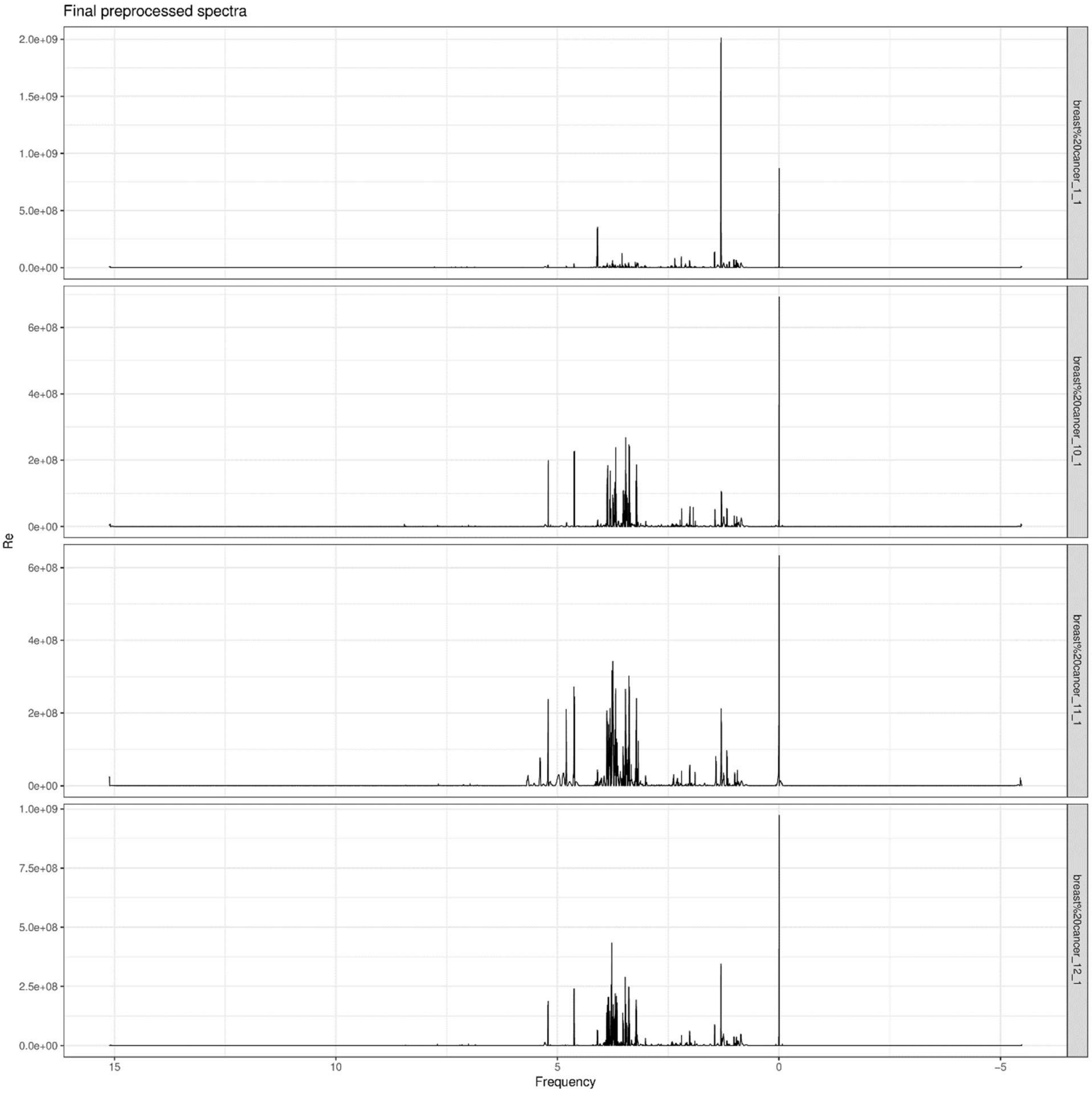

Figure S1 (a). Individual spectra (n = 46) from MetaboLights study MTBLS326 after being read and preprocessed through Workflows4Metabolomics. Samples Breast%20cancer_1_1, Breast%20cancer_10_1, Breast%20cancer_11_1, and Breast%20cancer_12_1.

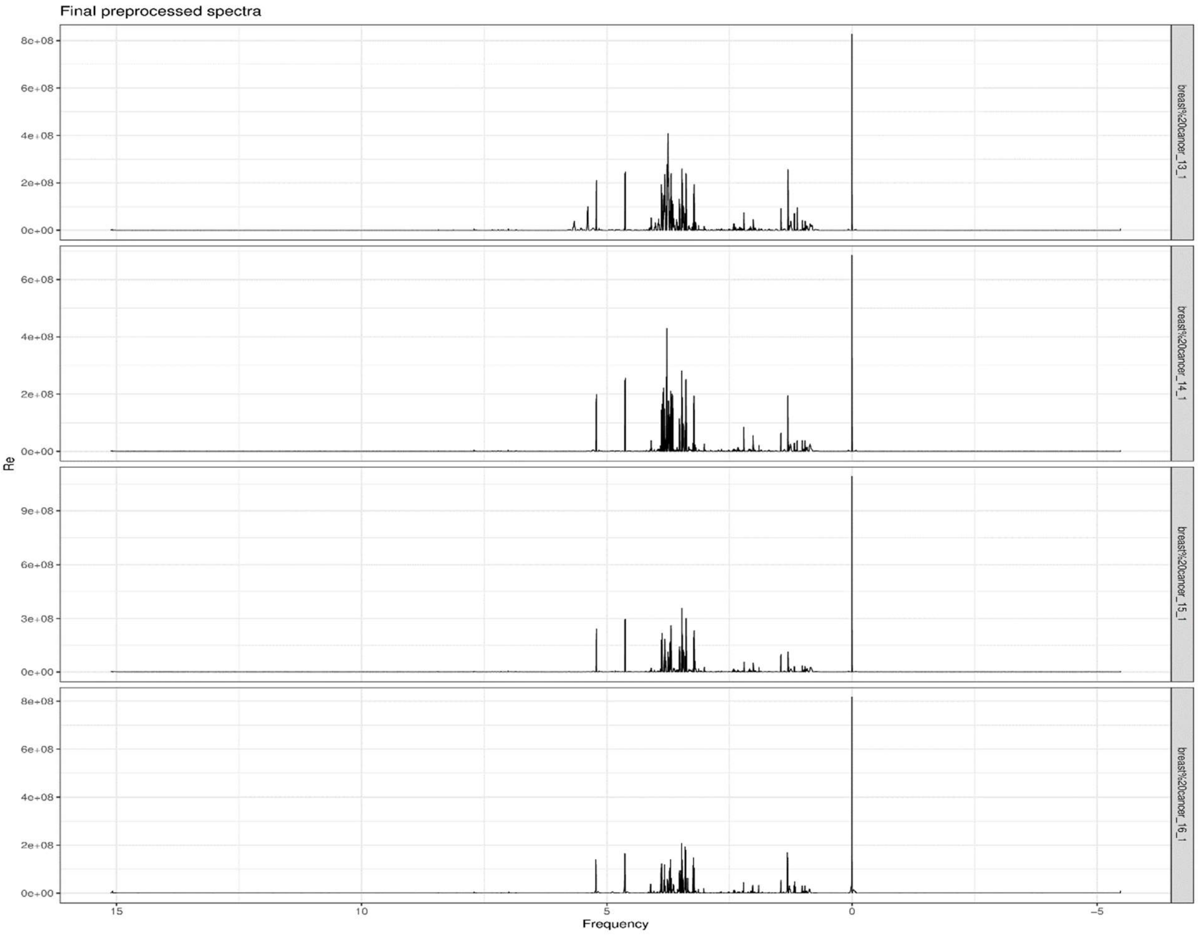

Figure S1 (b). Individual spectra (n = 46) from MetaboLights study MTBLS326 after being read and preprocessed through Workflows4Metabolomics. Samples Breast%20cancer_13_1, Breast%20cancer_14_1, Breast%20cancer_15_1, and Breast%20cancer_16_1.

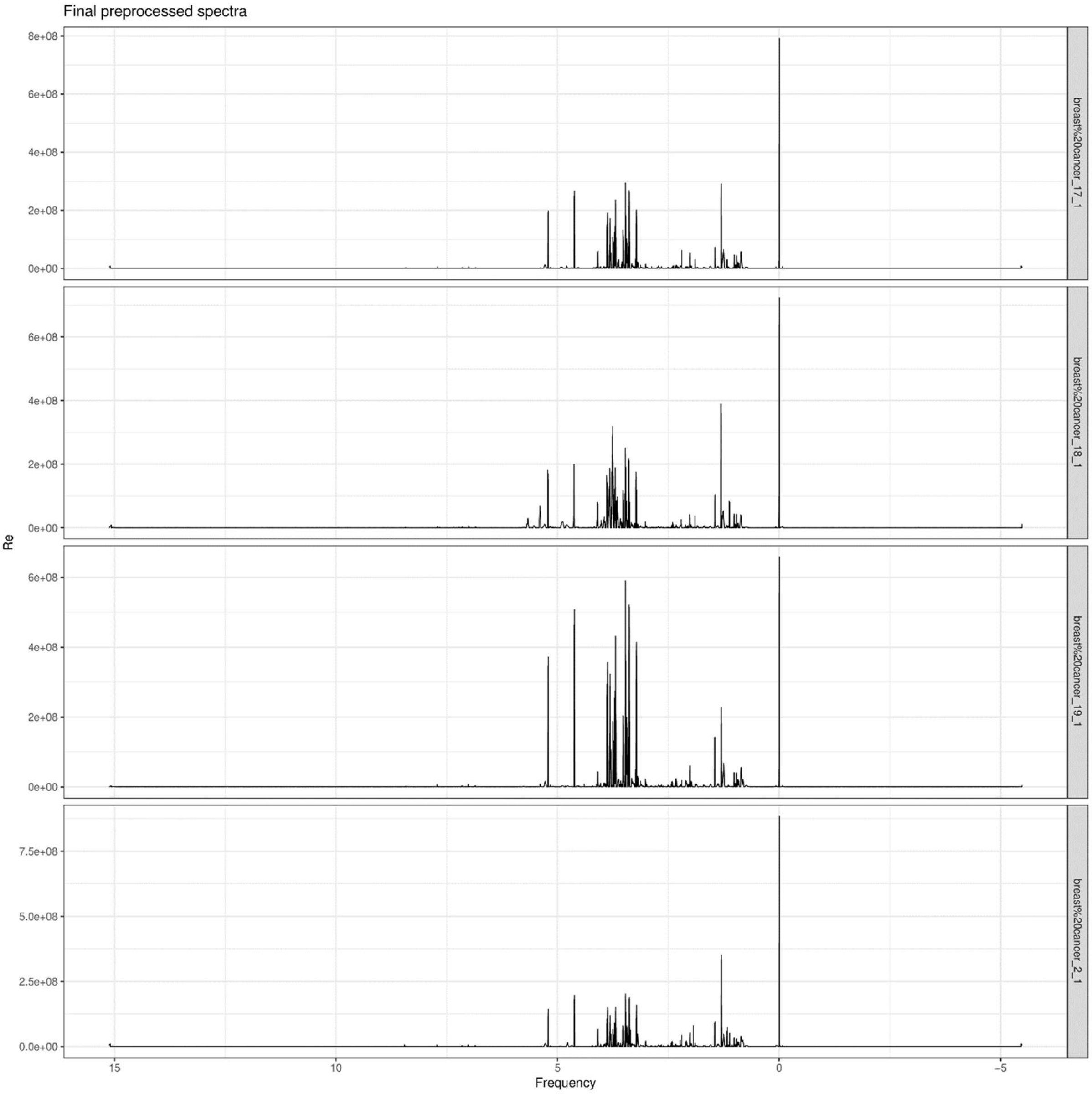

Figure S1 (c). Individual spectra (n = 46) from MetaboLights study MTBLS326 after being read and preprocessed through Workflows4Metabolomics. Samples Breast%20cancer_17_1, Breast%20cancer_18_1, Breast%20cancer_19_1, and Breast%20cancer_2_1.

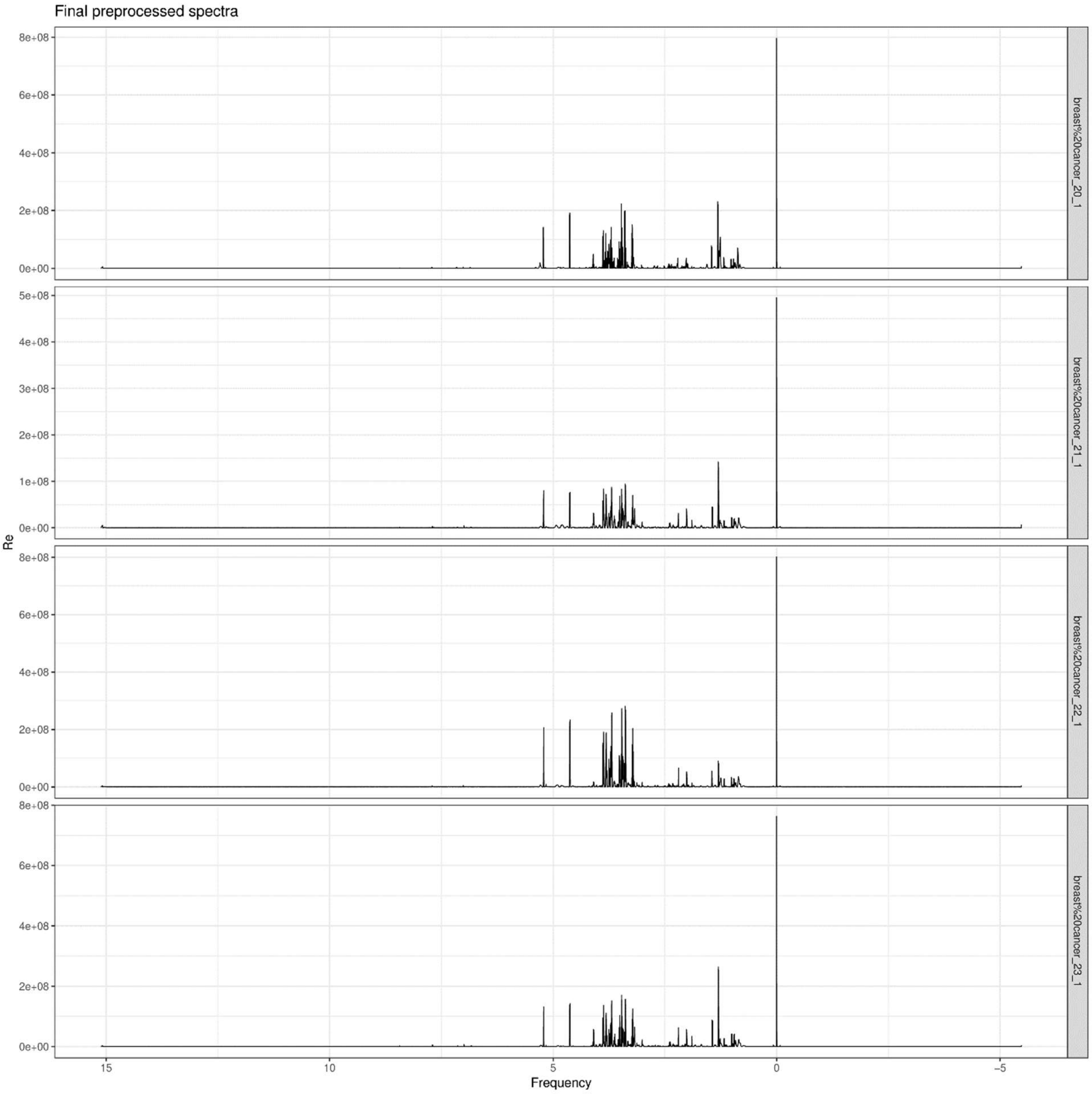

Figure S1 (d). Individual spectra (n = 46) from MetaboLights study MTBLS326 after being read and preprocessed through Workflows4Metabolomics. Samples Breast%20cancer_20_1, Breast%20cancer_21_1, Breast%20cancer_22_1, and Breast%20cancer_23_1.

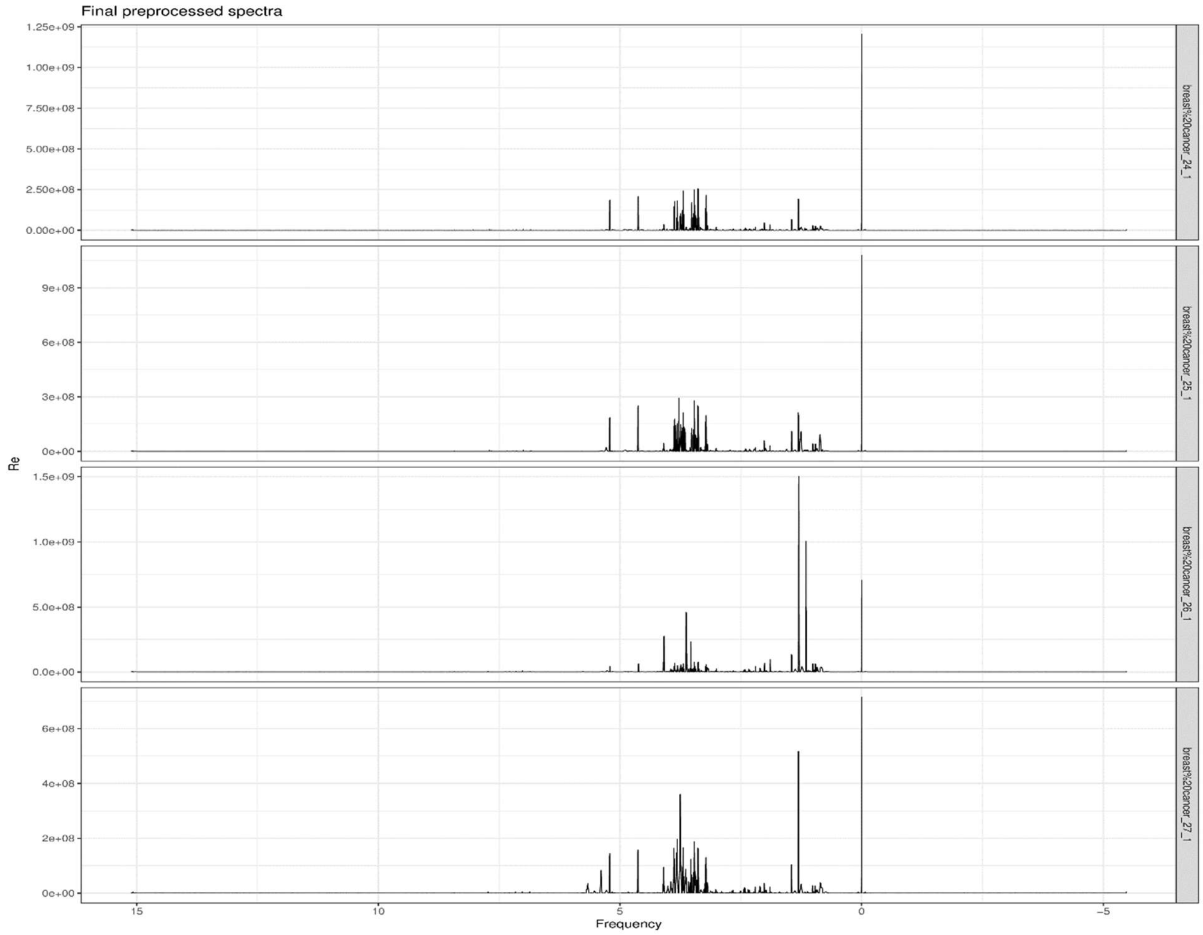

Figure S1 (e). Individual spectra (n = 46) from MetaboLights study MTBLS326 after being read and preprocessed through Workflows4Metabolomics. Samples Breast%20cancer_24_1, Breast%20cancer_25_1, Breast%20cancer_26_1, and Breast%20cancer_27_1.

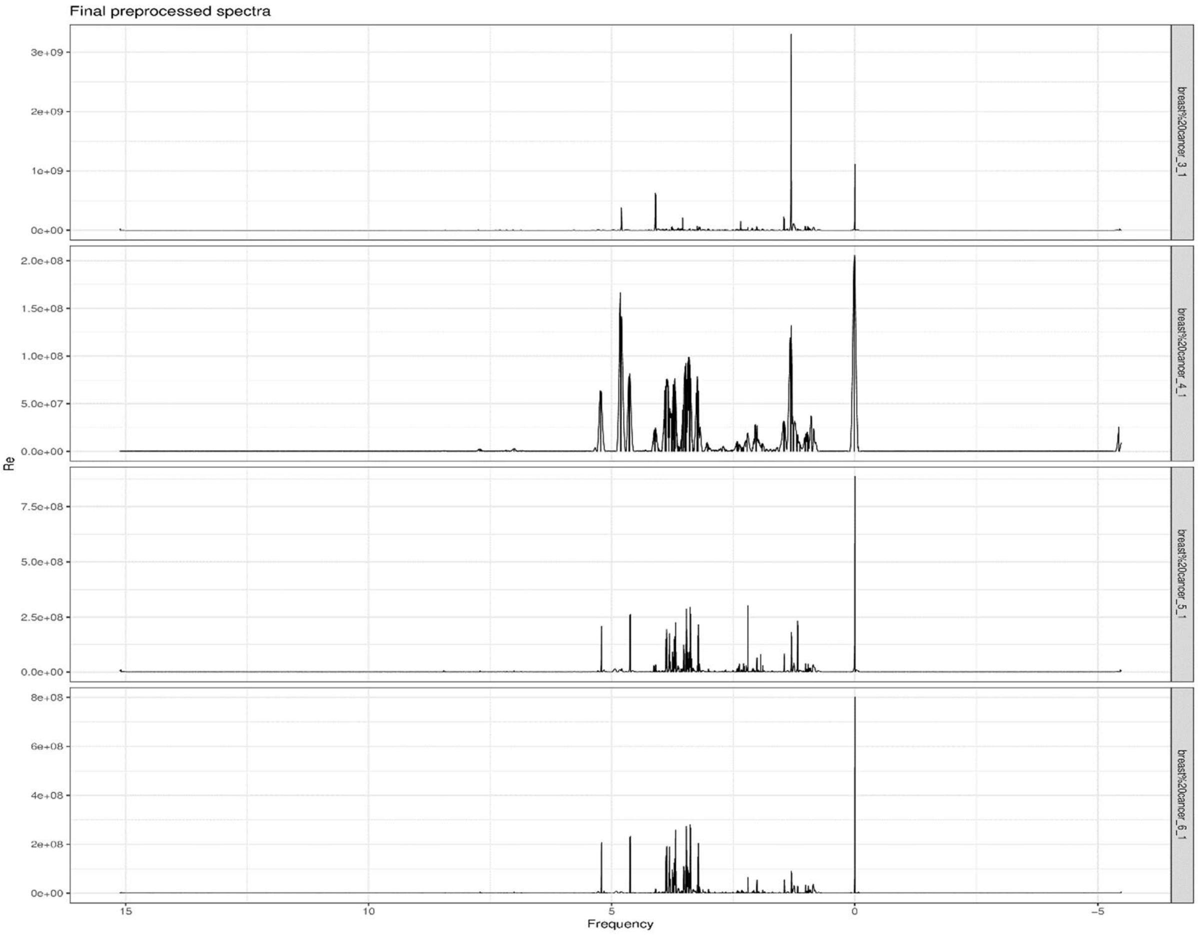

Figure S1 (f). Individual spectra (n = 46) from MetaboLights study MTBLS326 after being read and preprocessed through Workflows4Metabolomics. Samples Breast%20cancer_3_1, Breast%20cancer_4_1, Breast%20cancer_5_1, and Breast%20cancer_6_1.

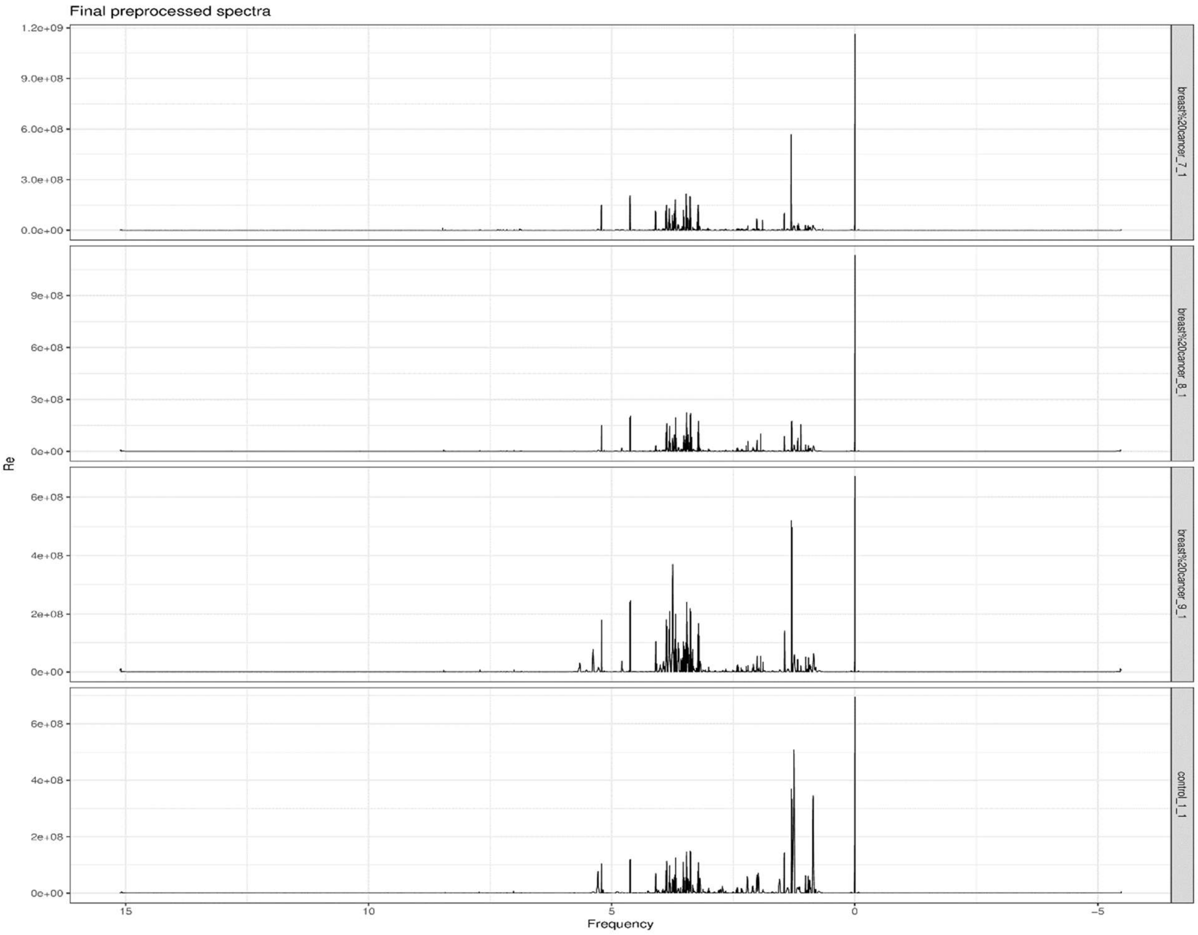

Figure S1 (g). Individual spectra (n = 46) from MetaboLights study MTBLS326 after being read and preprocessed through Workflows4Metabolomics. Samples Breast%20cancer_7_1, Breast%20cancer_8_1, Breast%20cancer_9_1, and control_1_1.

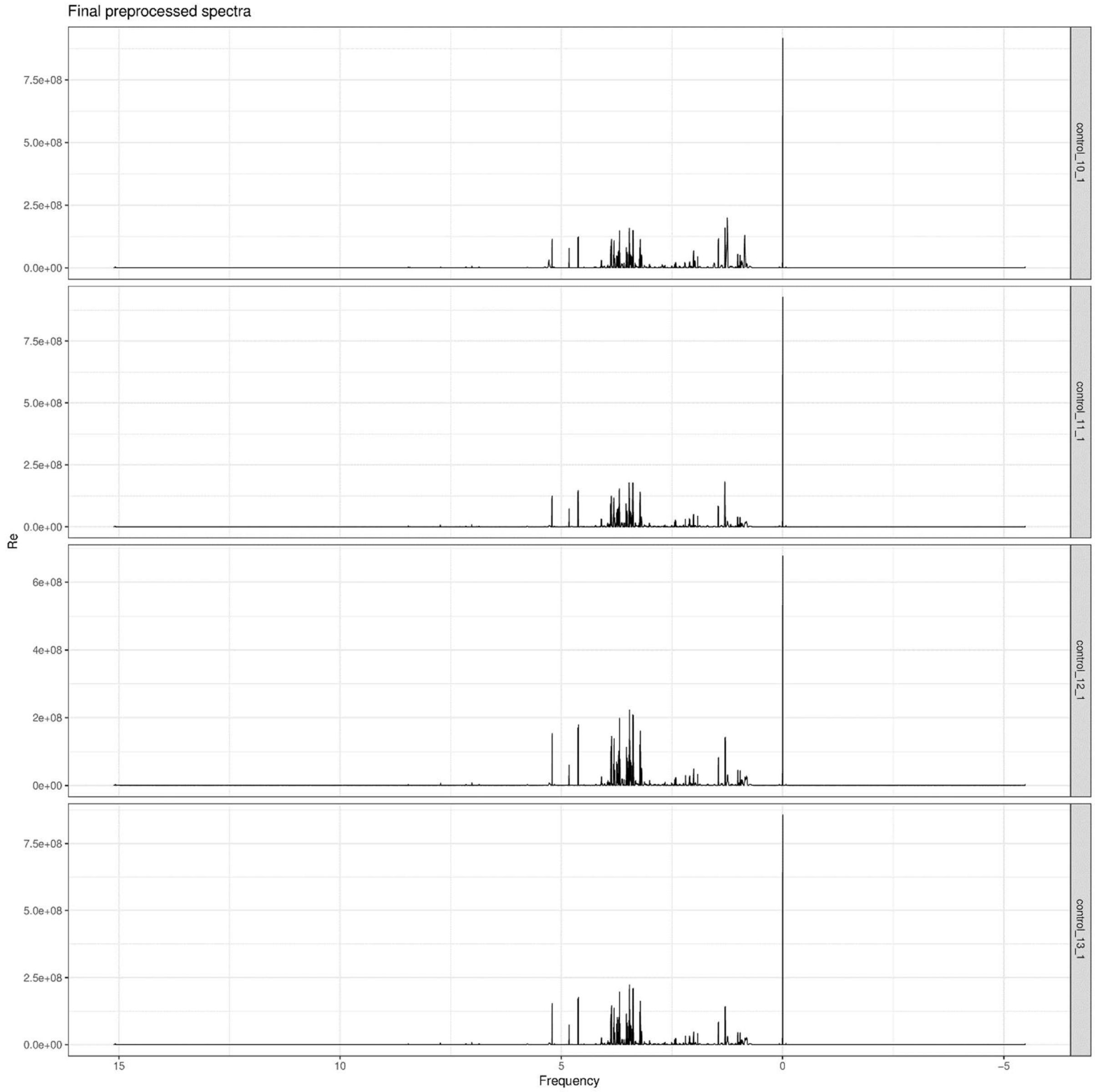

Figure S1 (h). Individual spectra (n = 46) from MetaboLights study MTBLS326 after being read and preprocessed through Workflows4Metabolomics. Samples control_10_1, control_11_1, control_12_1, and control_13_1.

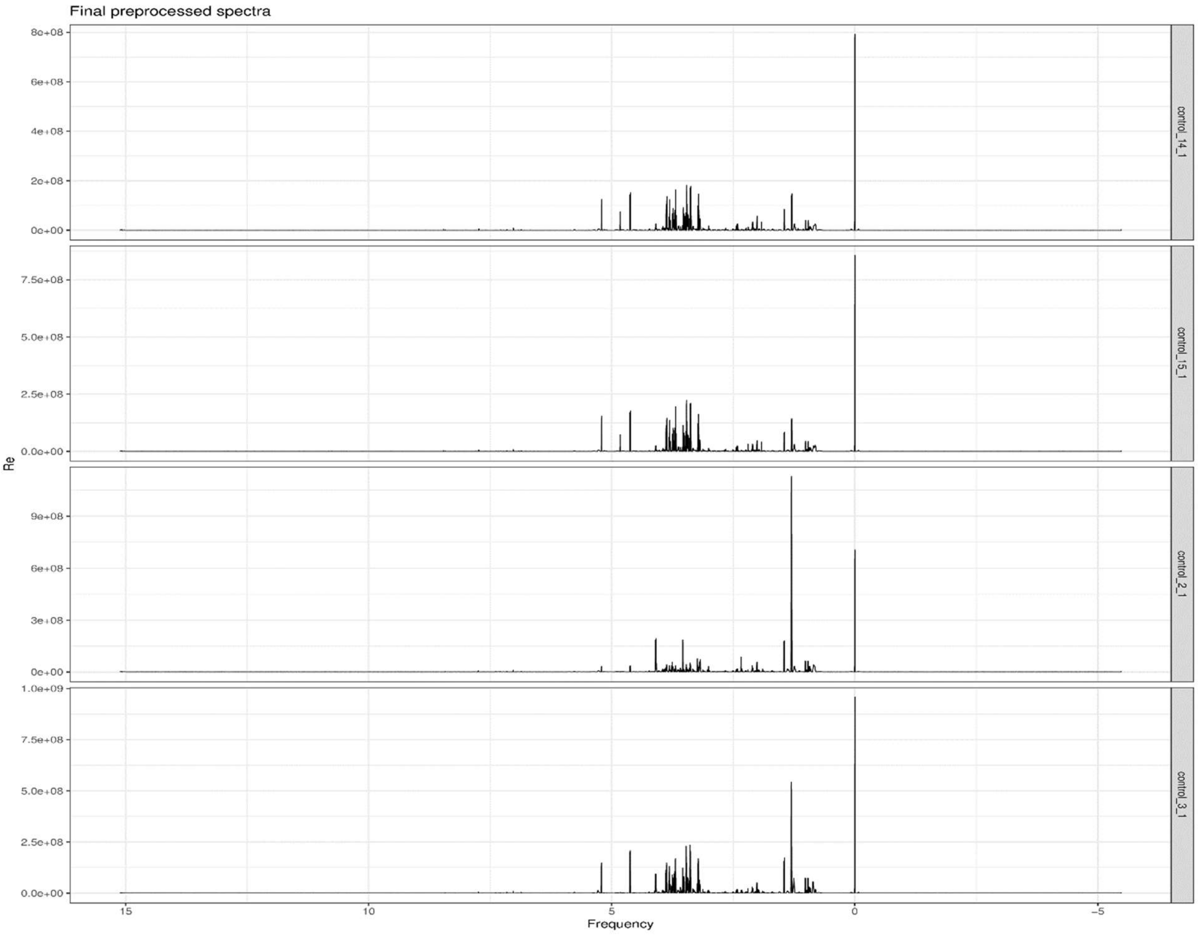

Figure S1 (i). Individual spectra (n = 46) from MetaboLights study MTBLS326 after being read and preprocessed through Workflows4Metabolomics. Samples control_14_1, control_15_1, control_2_1, and control_3_1.

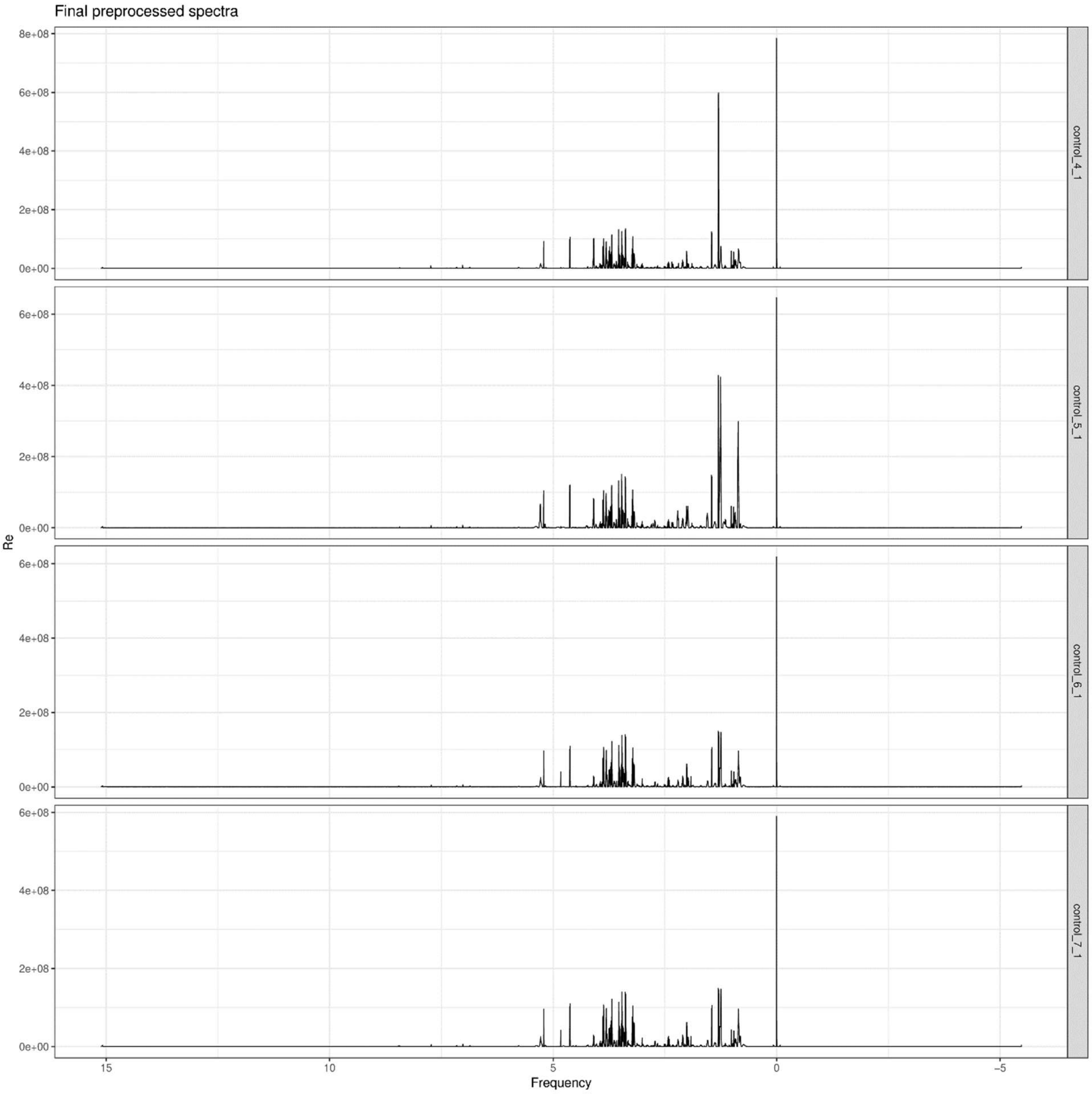

Figure S1 (j). Individual spectra (n = 46) from MetaboLights study MTBLS326 after being read and preprocessed through Workflows4Metabolomics. Samples control_4_1, control_5_1, control_6_1, and control_7_1.

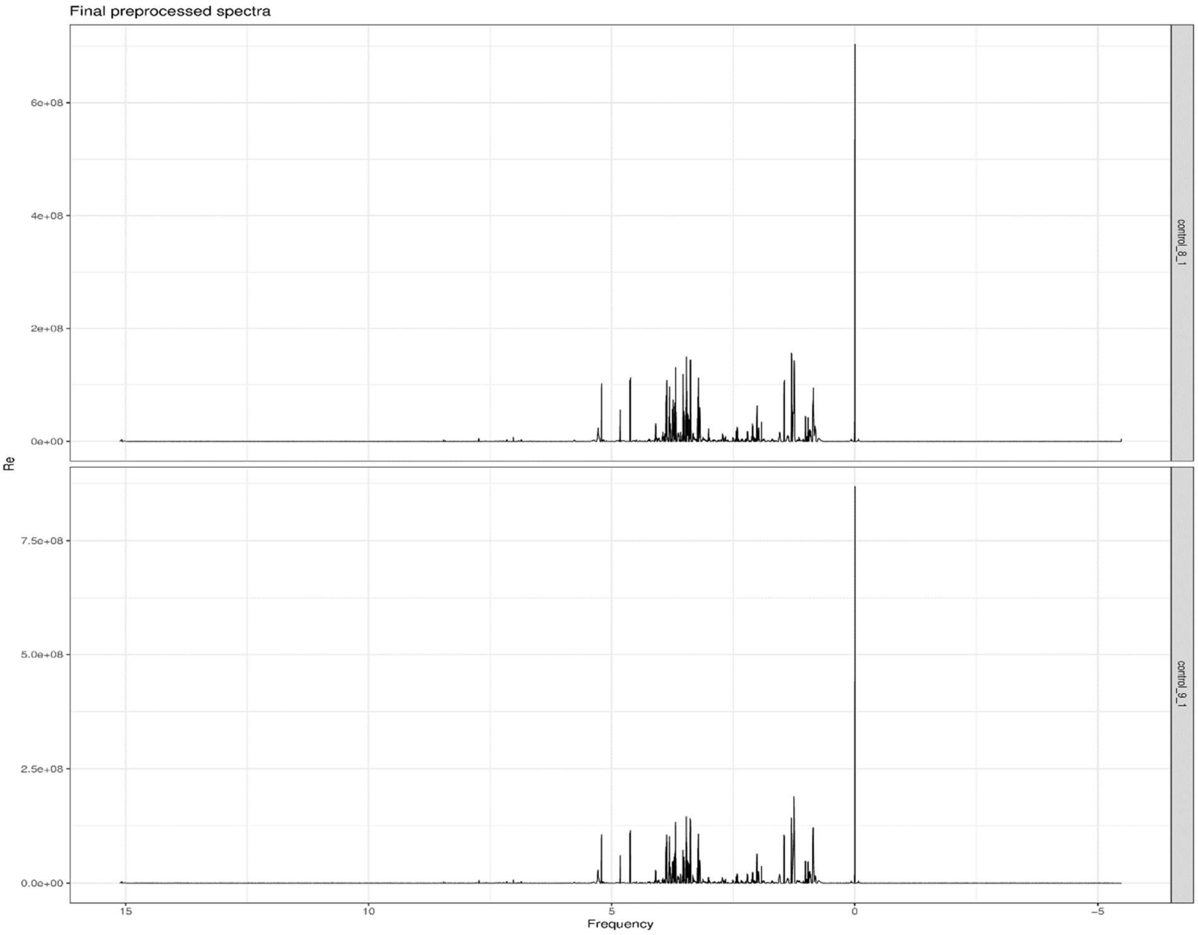

Figure S1 (k). Individual spectra (n = 46) from MetaboLights study MTBLS326 after being read and preprocessed through Workflows4Metabolomics. Samples control_8_1, and control_9_1.

MetaboLights study MTBLS431

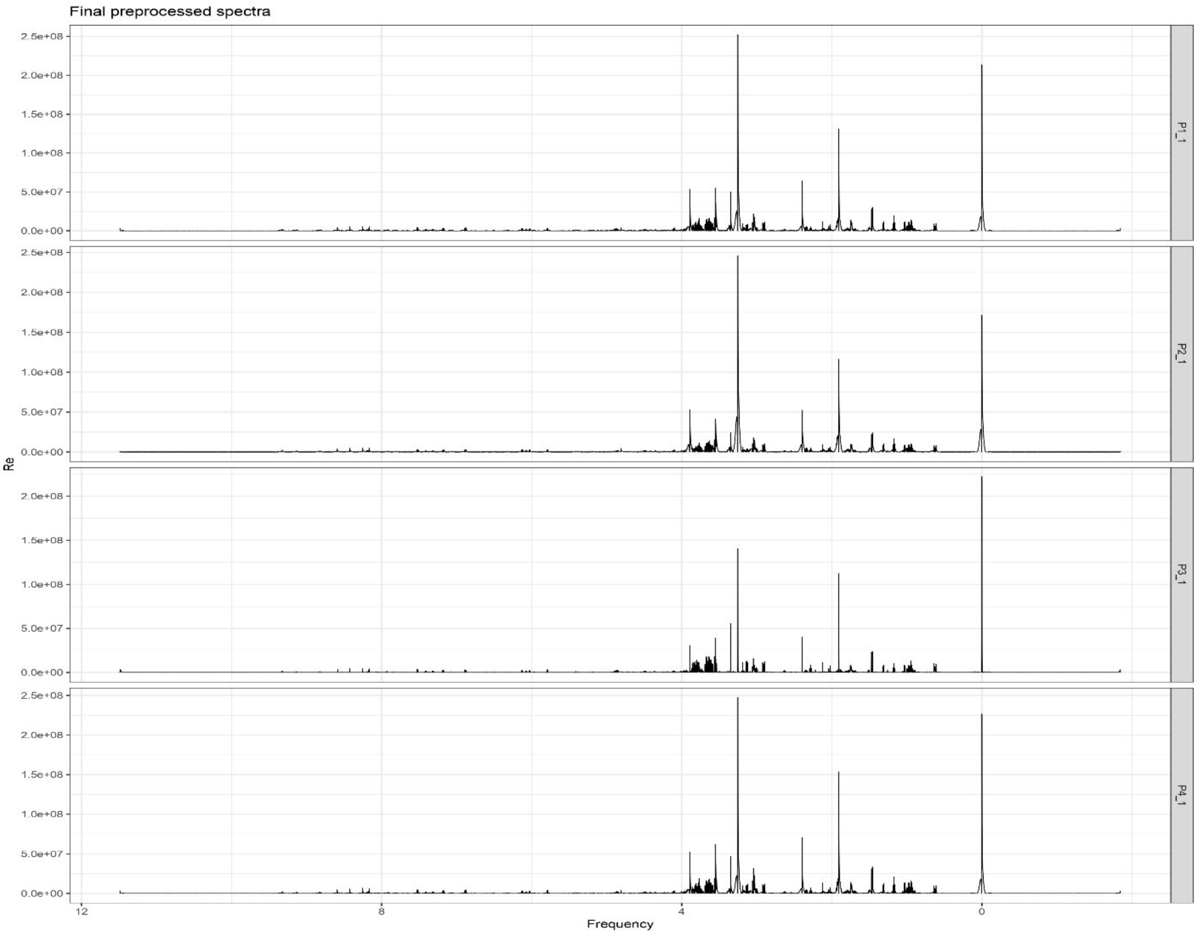

Figure S2 (a). Individual spectra (n = 10) from MetaboLights study MTBLS431 after being read and preprocessed through Workflows4Metabolomics. Samples P1_1, P2_1, P3_1, and P4_1.

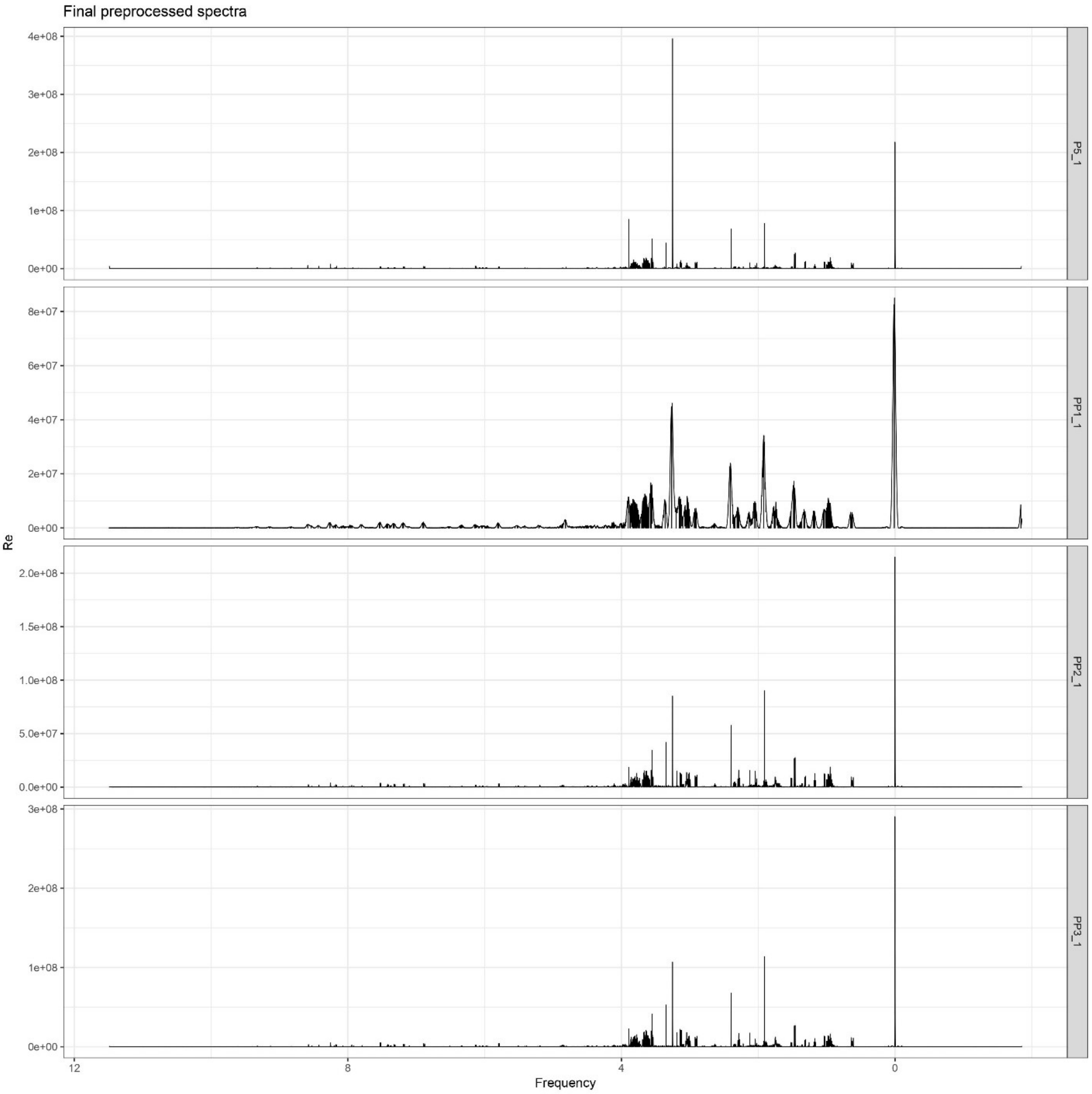

Figure S2 (b). Individual spectra (n = 10) from MetaboLights study MTBLS431 after being read and preprocessed through Workflows4Metabolomics. Samples P5_1, PP1_1, PP2_1, and PP3_1.

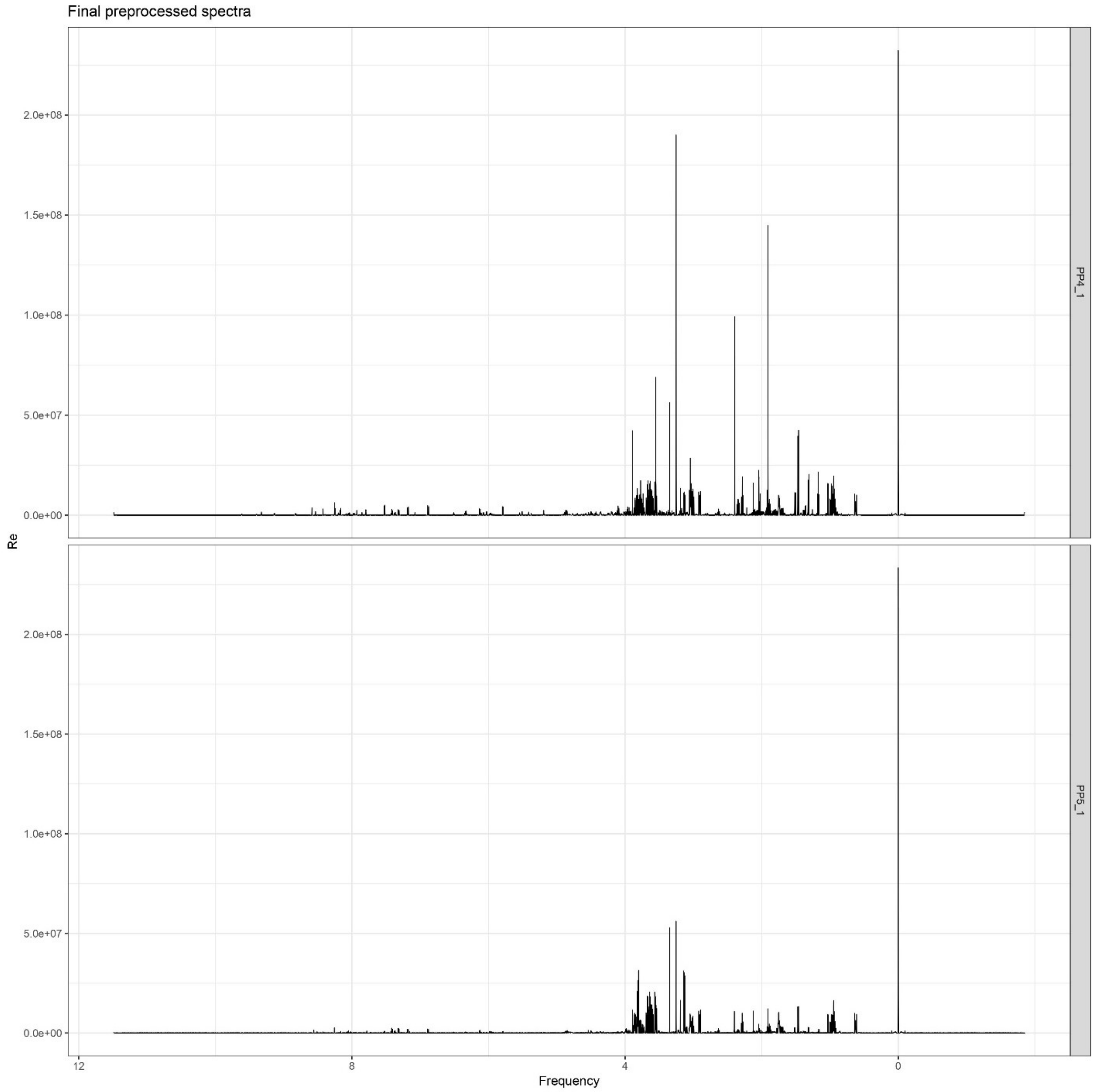

Figure S2 (c). Individual spectra (n = 10) from MetaboLights study MTBLS431 after being read and preprocessed through Workflows4Metabolomics. Samples PP4_1, and PP5_1.

MetaboLights study MTBLS869

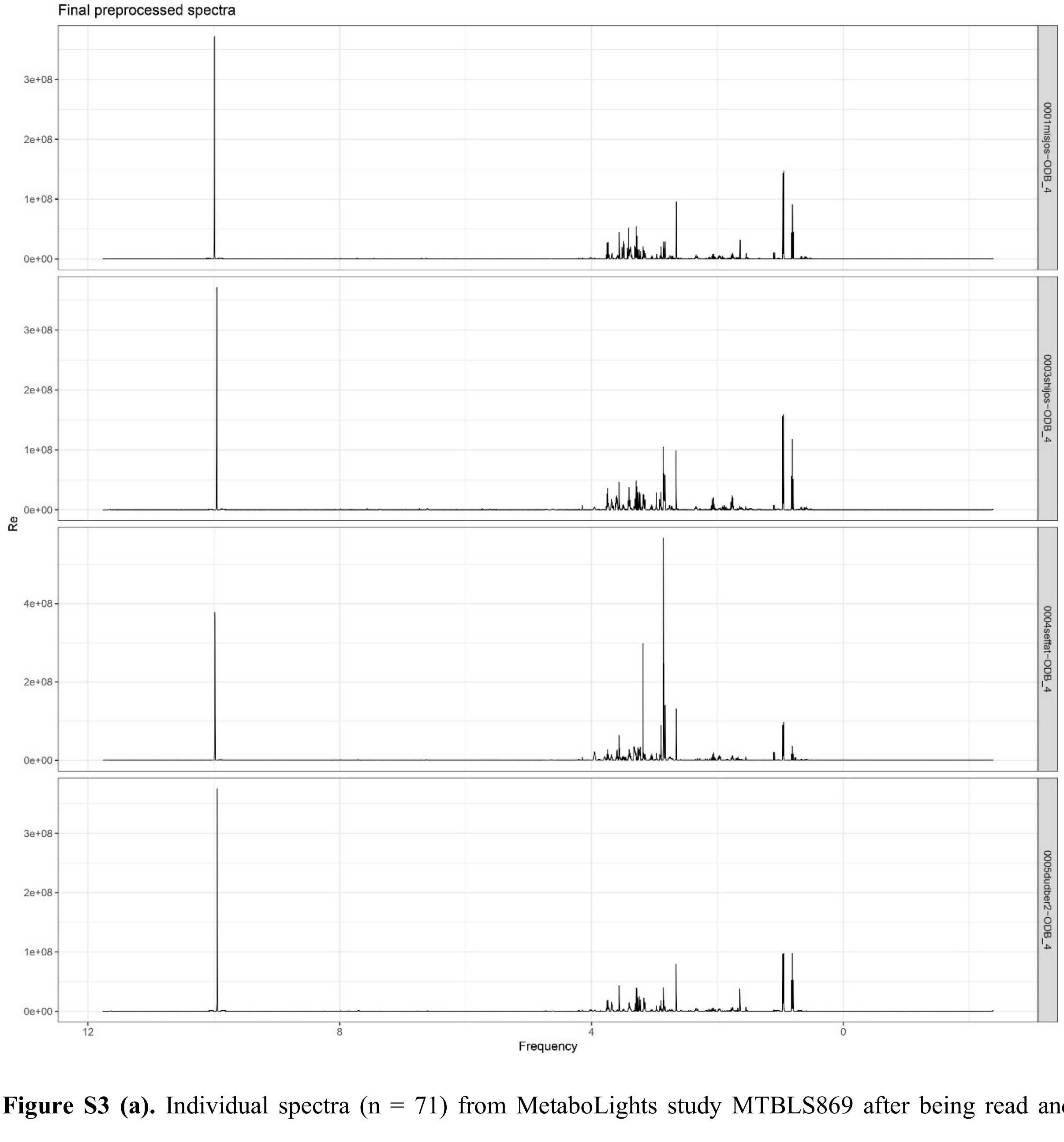

Figure S3 (a). Individual spectra (n = 71) from MetaboLights study MTBLS869 after being read and preprocessed through Workflows4Metabolomics. Samples 0001misjos-ODB_4, 0003shijos-ODB_4, 0004seffat-ODB_4, and 0005dudber2-ODB_4.

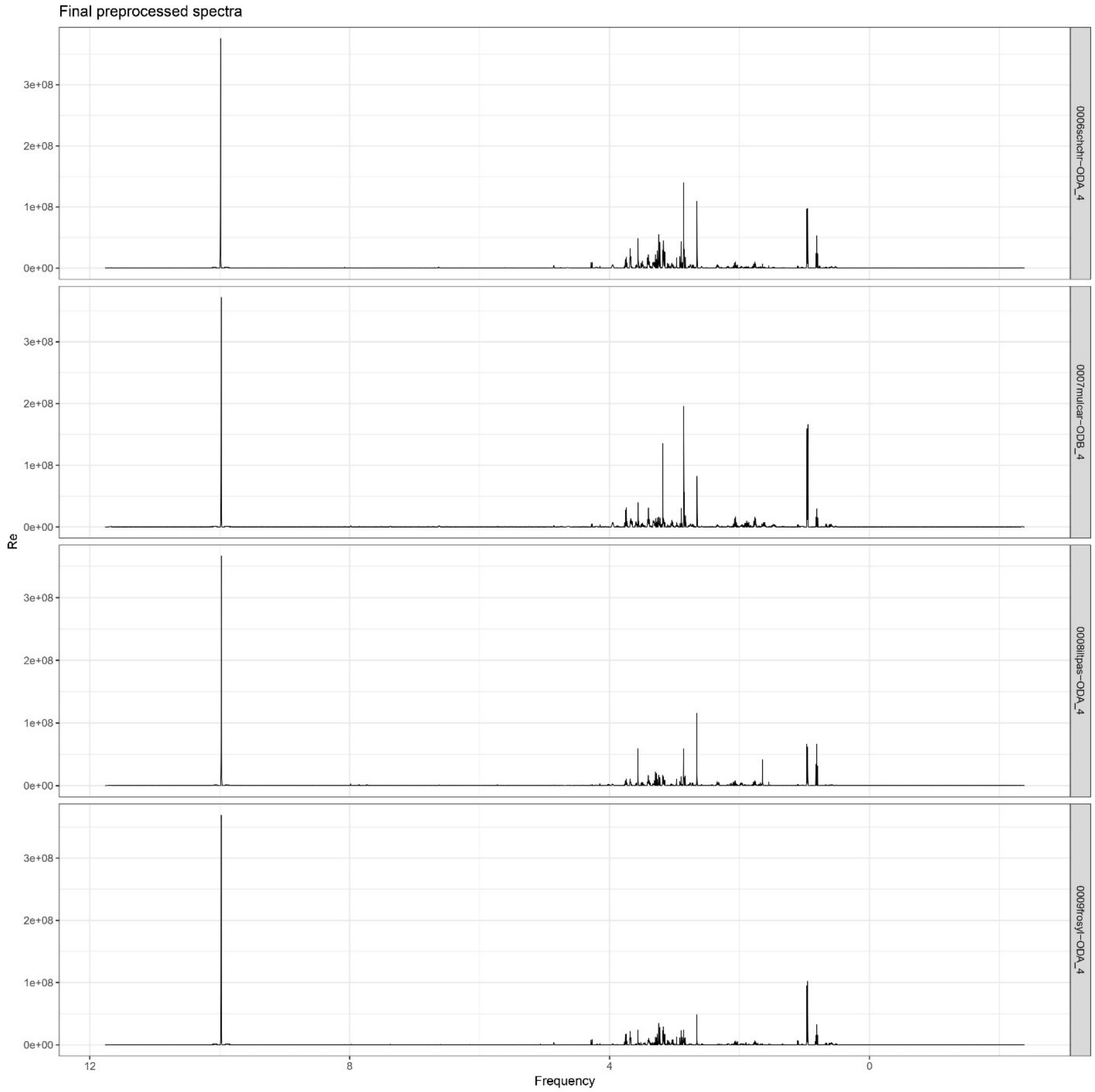

Figure S3 (b). Individual spectra (n = 71) from MetaboLights study MTBLS869 after being read and preprocessed through Workflows4Metabolomics. Samples 0006schchr-ODA_4, 0007mulcar-ODB_4, 0008iltpas-ODA_4, and 0009frosyl-ODA_4.

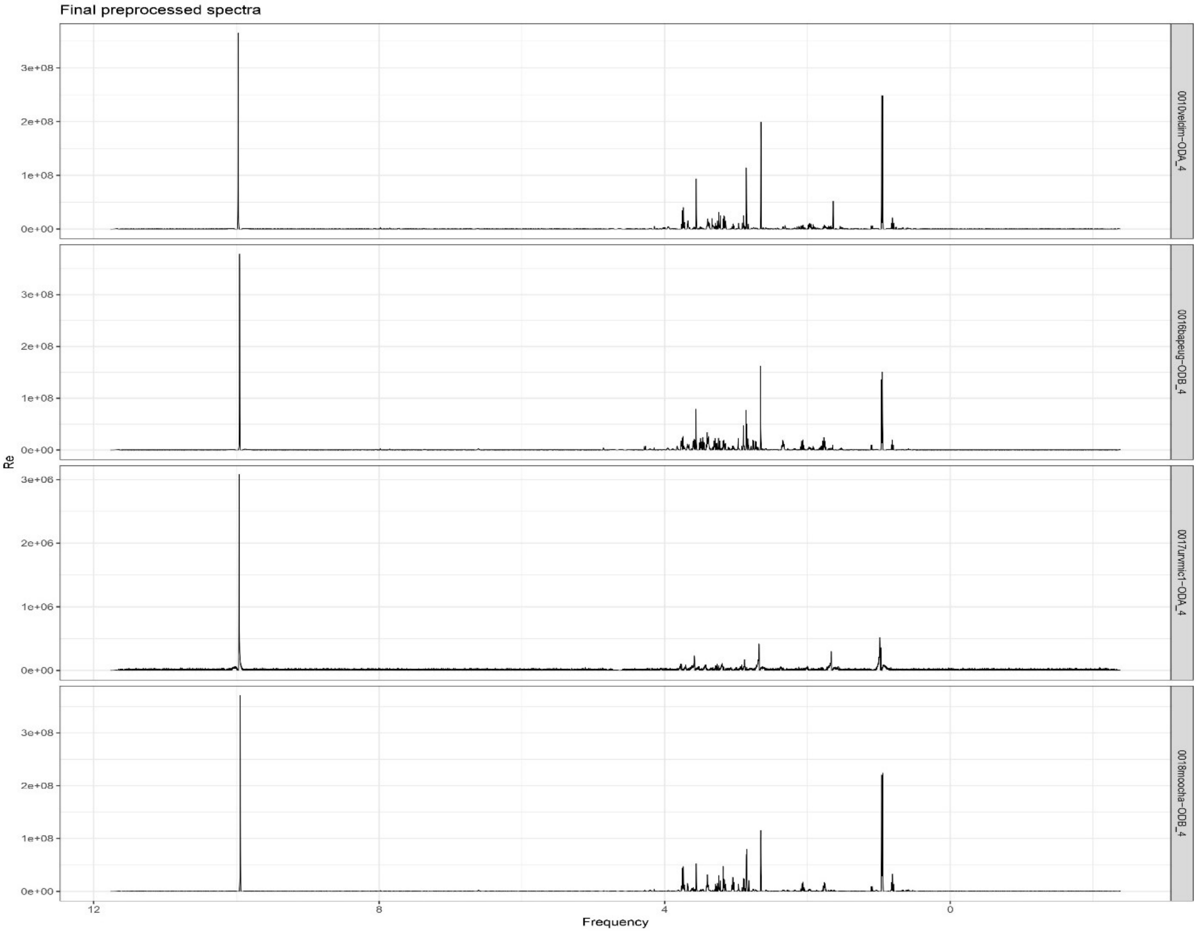

Figure S3 (c). Individual spectra (n = 71) from MetaboLights study MTBLS869 after being read and preprocessed through Workflows4Metabolomics. Samples 0010veldim-ODA_4, 0016bapeug-ODB_4, 0017urevmic1-ODA_4, and 0018mcocha-ODB_4.

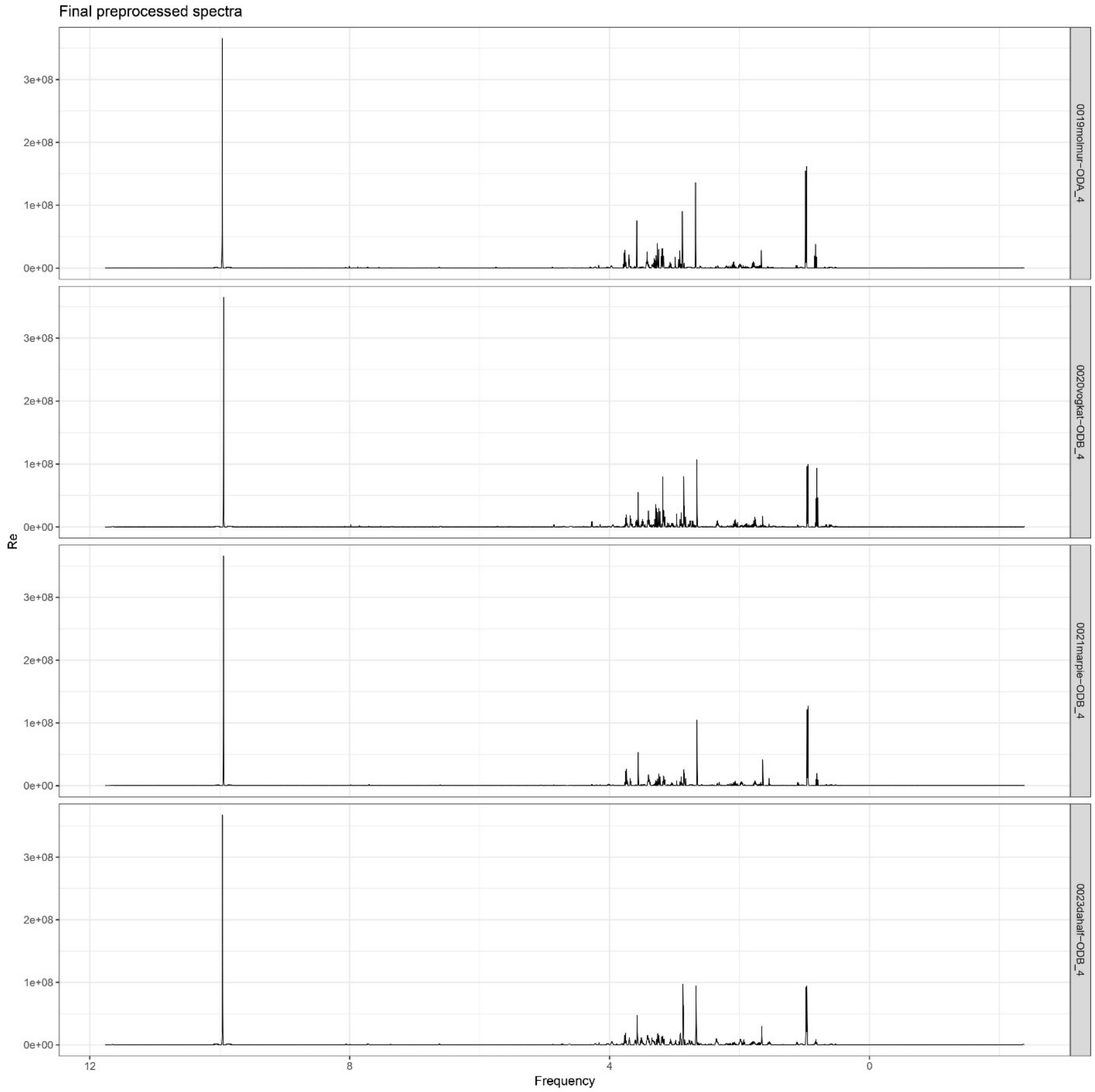

Figure S3 (d). Individual spectra (n = 71) from MetaboLights study MTBLS869 after being read and preprocessed through Workflows4Metabolomics. Samples 0019molmur-ODA_4, 0020vogkat-ODB_4, 0021marpie-ODB_4, and 0023dahalf-ODB_4.

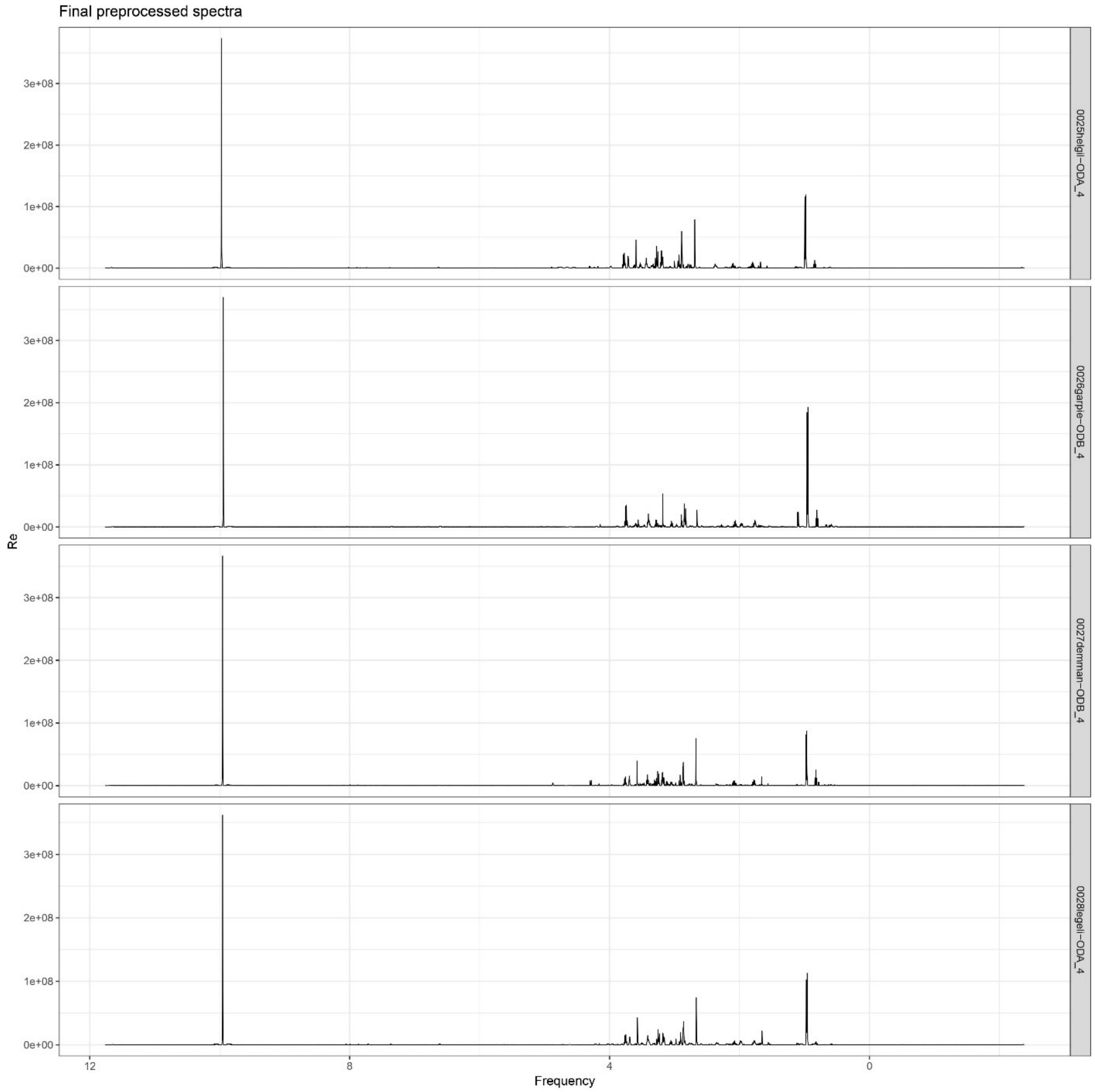

Figure S3 (e). Individual spectra (n = 71) from MetaboLights study MTBLS869 after being read and preprocessed through Workflows4Metabolomics. Samples 0025helgil-ODA_4, 0026gareie-ODB_4, 0027demman-ODB_4, and 0028legeli-ODA_4.

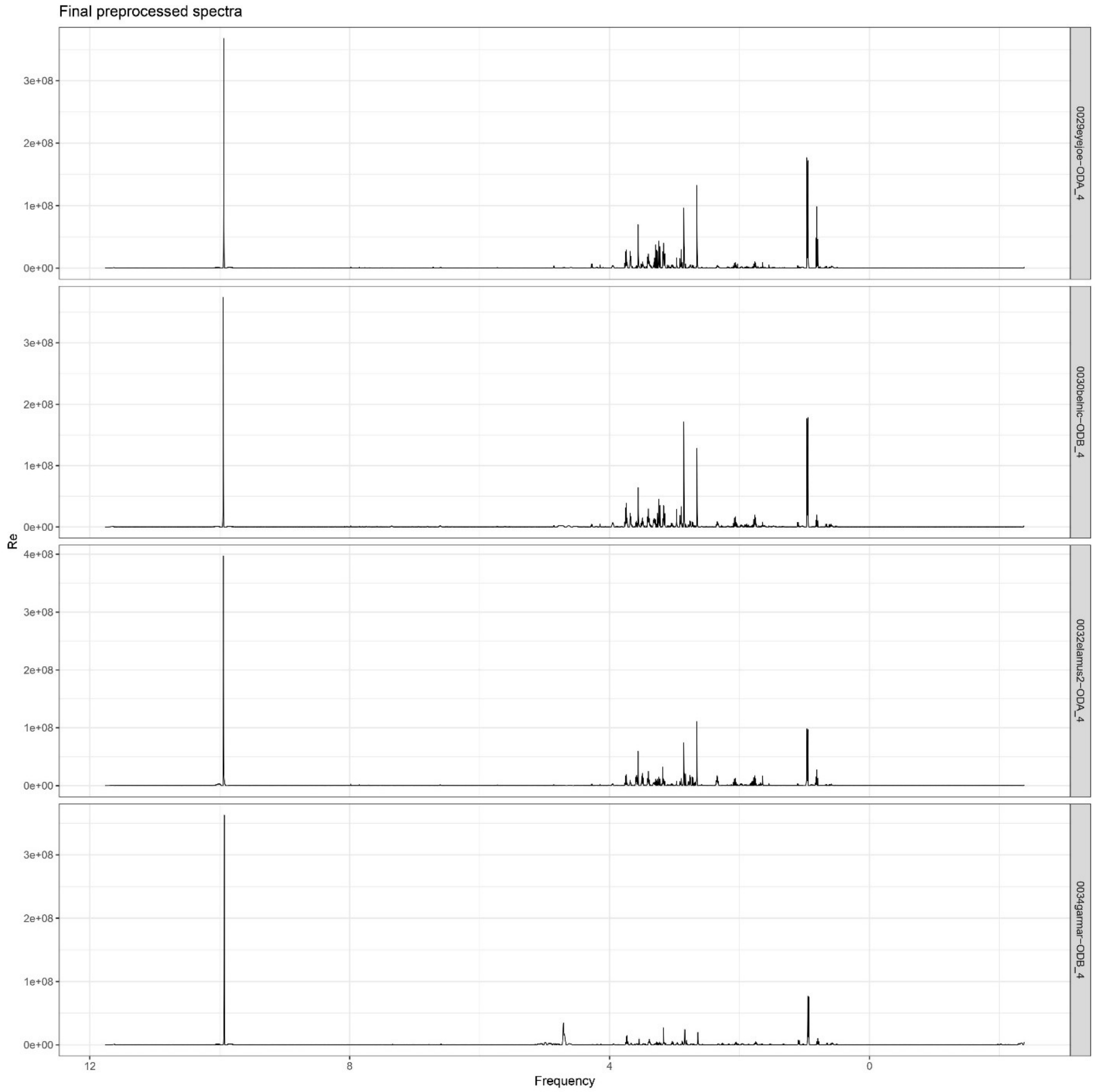

Figure S3 (f). Individual spectra (n = 71) from MetaboLights study MTBLS869 after being read and preprocessed through Workflows4Metabolomics. Samples 0029eyejoe-ODA_4, 0030belnic-ODB_4, 0032elamus2-ODA_4, and 0034garmar-ODB_4.

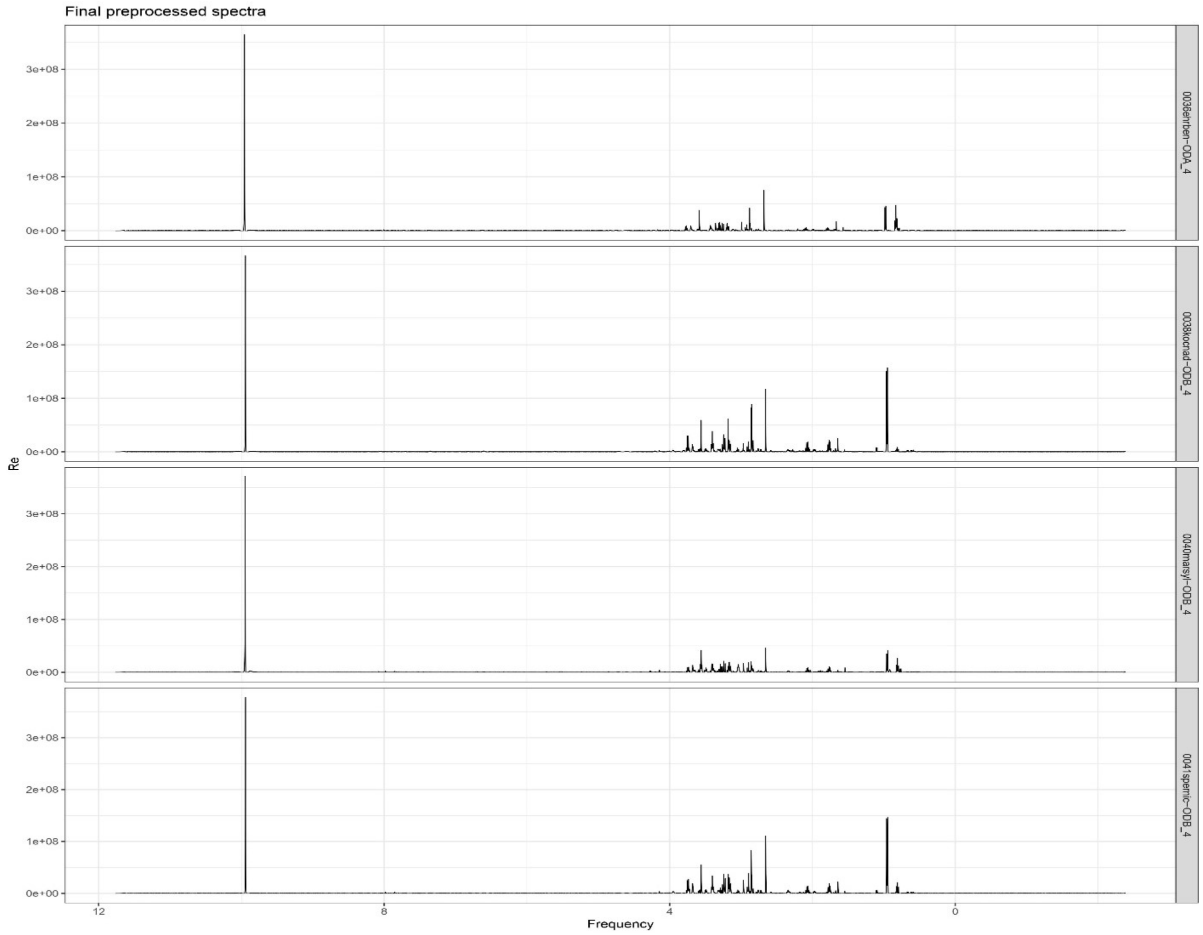

Figure S3 (g). Individual spectra (n = 71) from MetaboLights study MTBLS869 after being read and preprocessed through Workflows4Metabolomics. Samples 0036ehrben-ODA_4, 0038kocnad-ODB_4, 0040marsyl-ODB_4, and 0041spemic-ODB_4.

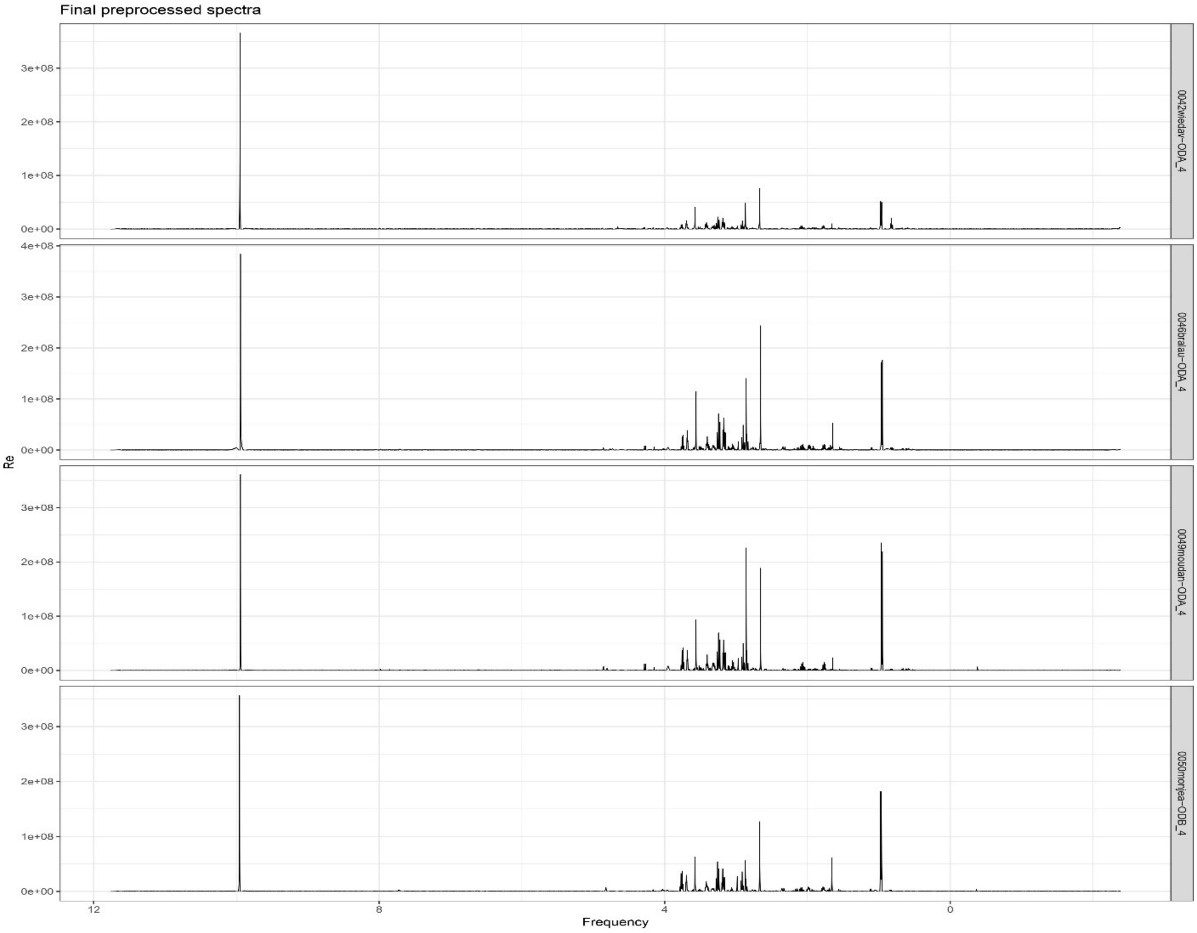

Figure S3 (h). Individual spectra (n = 71) from MetaboLights study MTBLS869 after being read and preprocessed through Workflows4Metabolomics. Samples 0042wiedav-ODA_4, 0046bralau-ODA_4, 0049moudan-ODA_4, and 0050monjea-ODB_4.

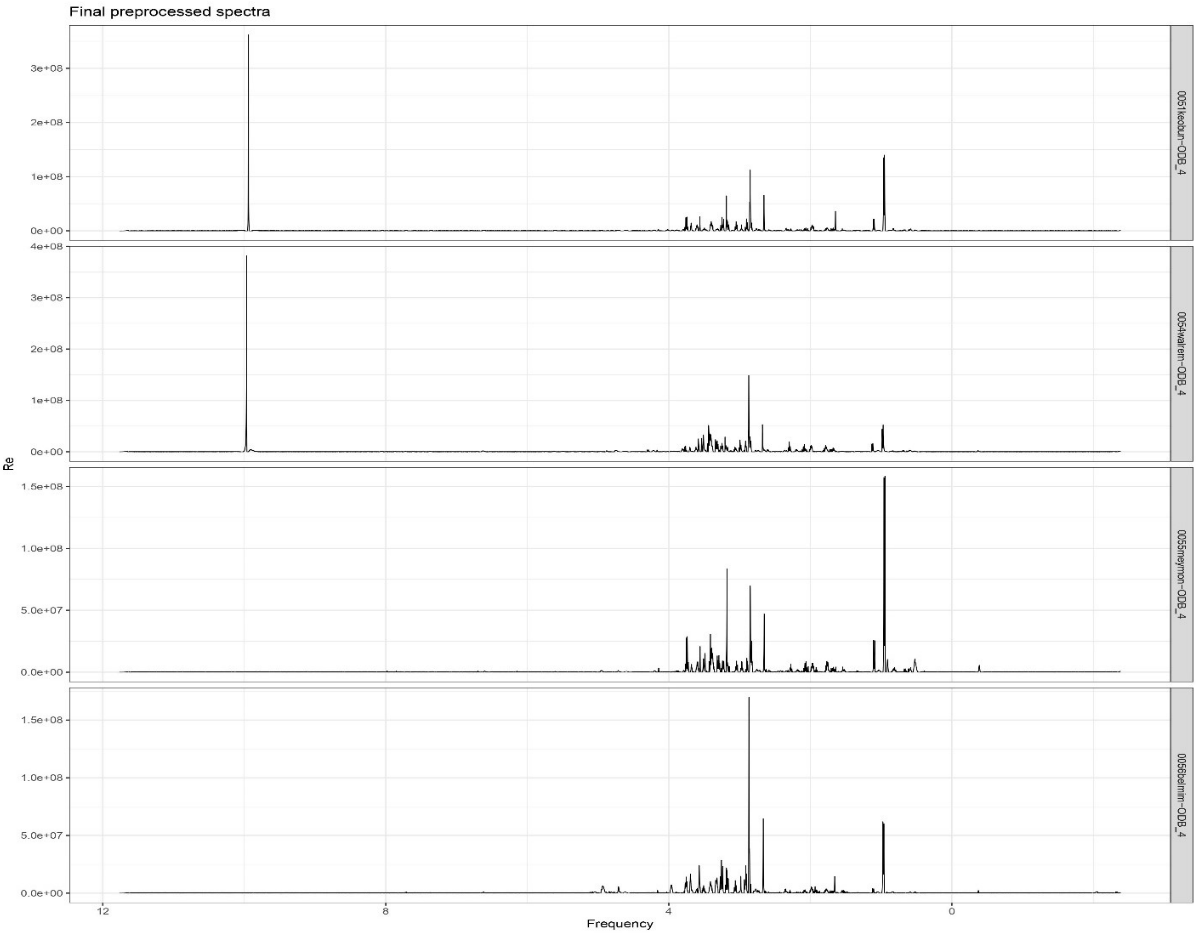

Figure S3 (i). Individual spectra (n = 71) from MetaboLights study MTBLS869 after being read and preprocessed through Workflows4Metabolomics. Samples 0051keobun-ODB_4, 0054walrem-ODB_4, 0055meymon-ODB_4, and 0056belmim-ODB_4.

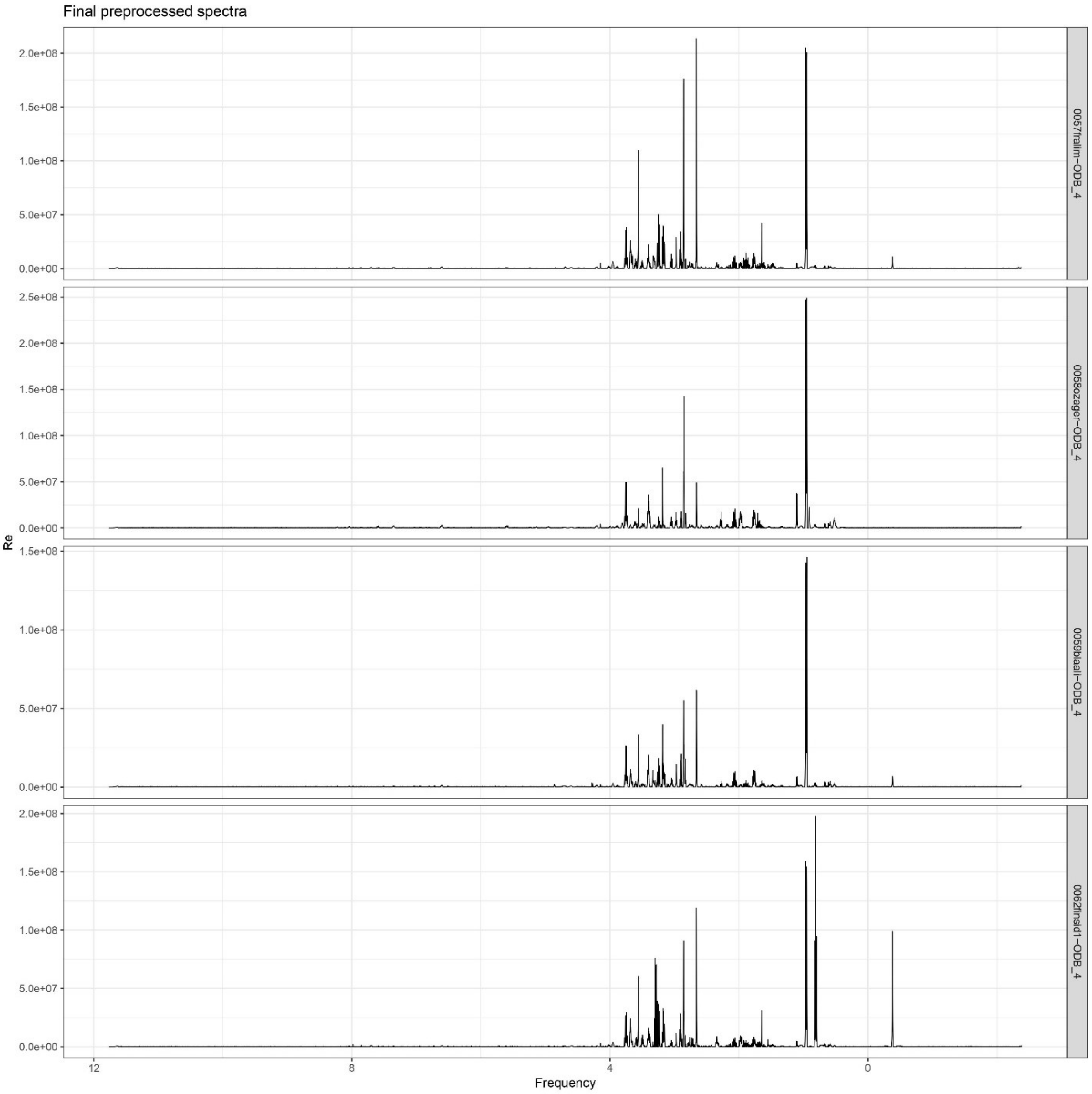

Figure S3 (j). Individual spectra (n = 71) from MetaboLights study MTBLS869 after being read and preprocessed through Workflows4Metabolomics. Samples 0057fralim-ODB_4, 0058ozager-ODB_4, 0059blaali-ODB_4, and 0062finsid1-ODB_4.

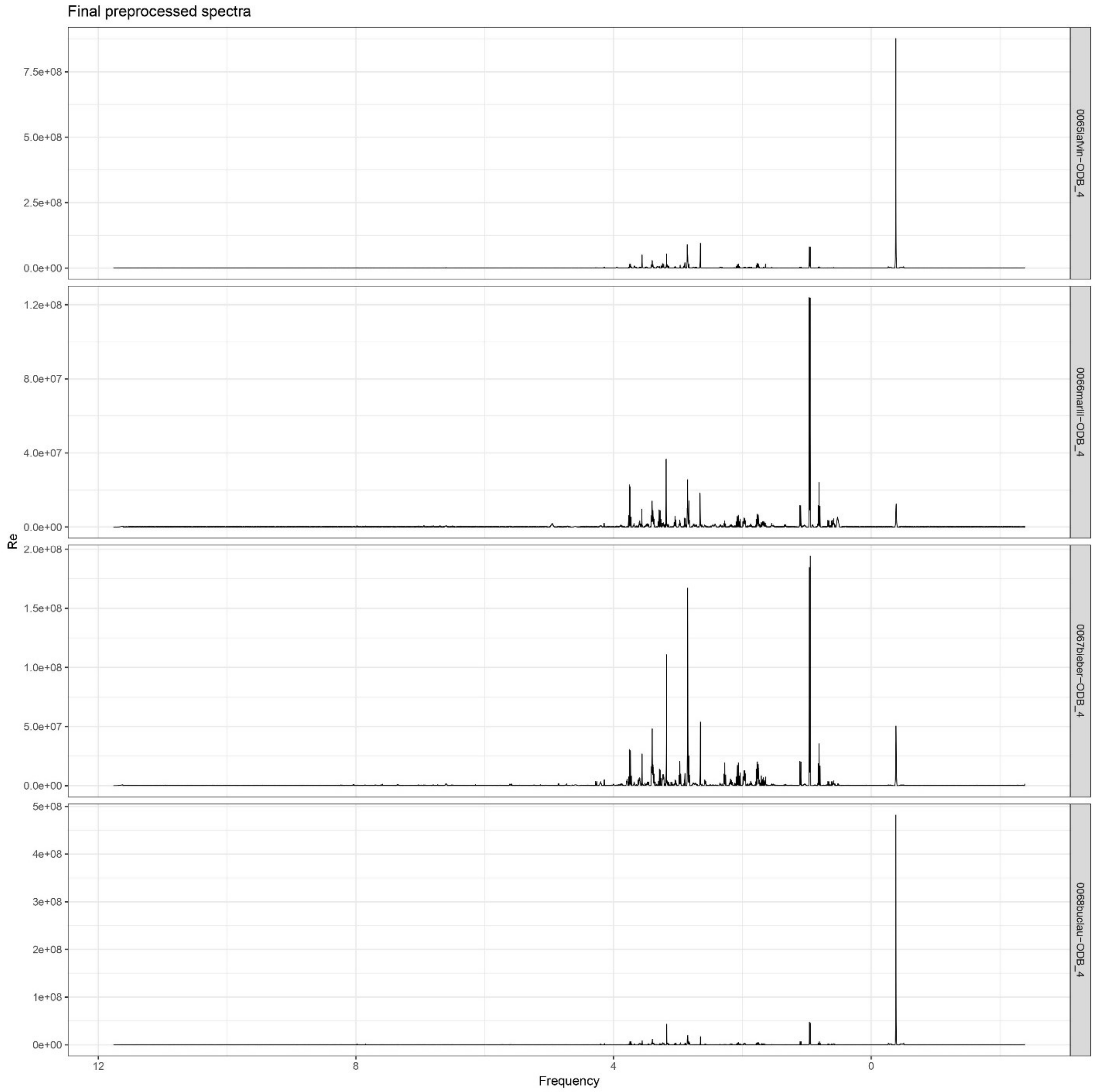

Figure S3 (k). Individual spectra (n = 71) from MetaboLights study MTBLS869 after being read and preprocessed through Workflows4Metabolomics. Samples 0065lafvin-ODB_4, 0066marlil-ODB_4, 0067bieber-ODB_4, and 0068buclau-ODB_4.

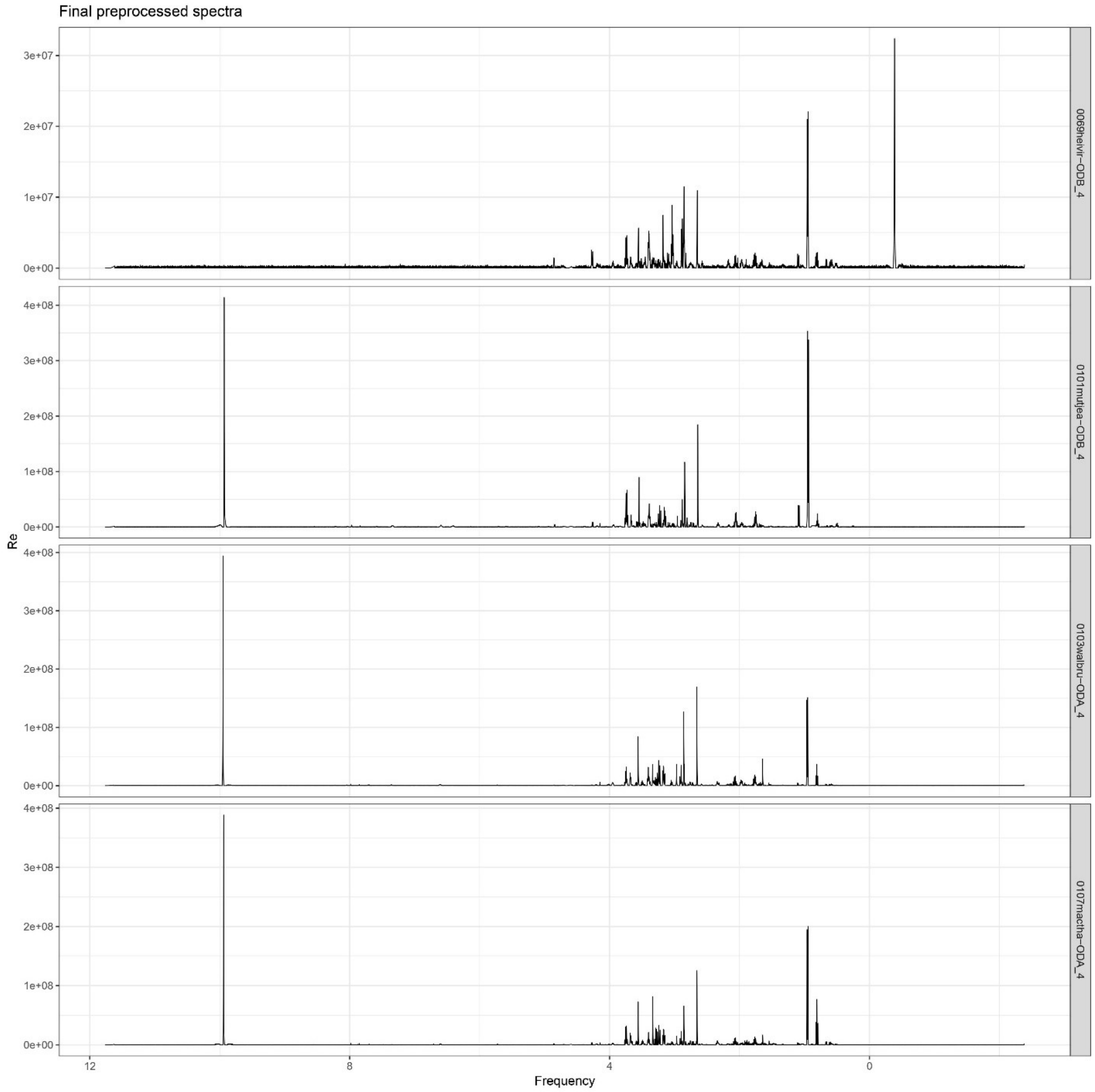

Figure S3 (l). Individual spectra (n = 71) from MetaboLights study MTBLS869 after being read and preprocessed through Workflows4Metabolomics. Samples 0069heivir-ODB_4, 0101mutjea-ODB_4, 0103walbru-ODA_4, and 0107mactha-ODA_4.

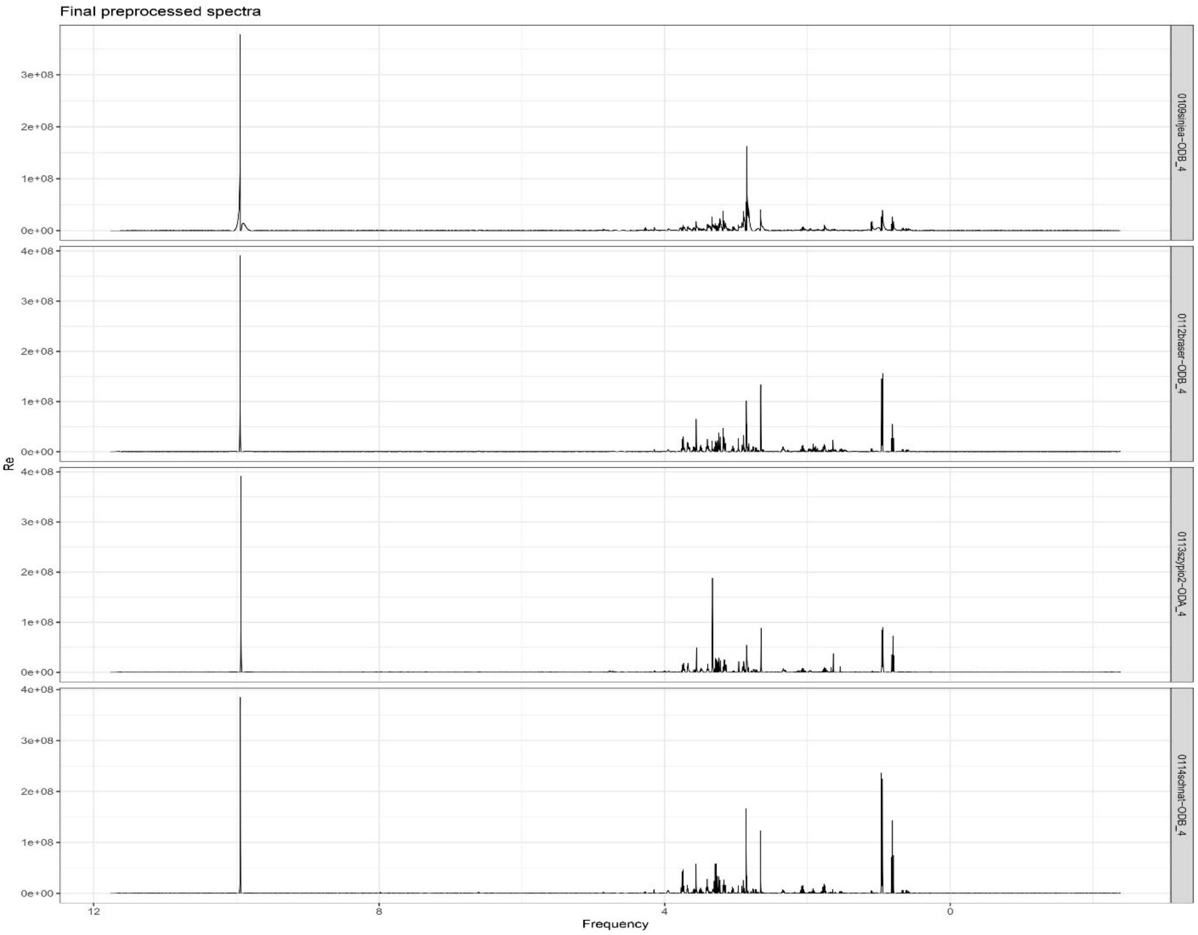

Figure S3 (m). Individual spectra (n = 71) from MetaboLights study MTBLS869 after being read and preprocessed through Workflows4Metabolomics. Samples 0109sinjea-ODB_4, 0112braser-ODB_4, 0113szypio2-ODA_4, and 0114schnat-ODB_4.

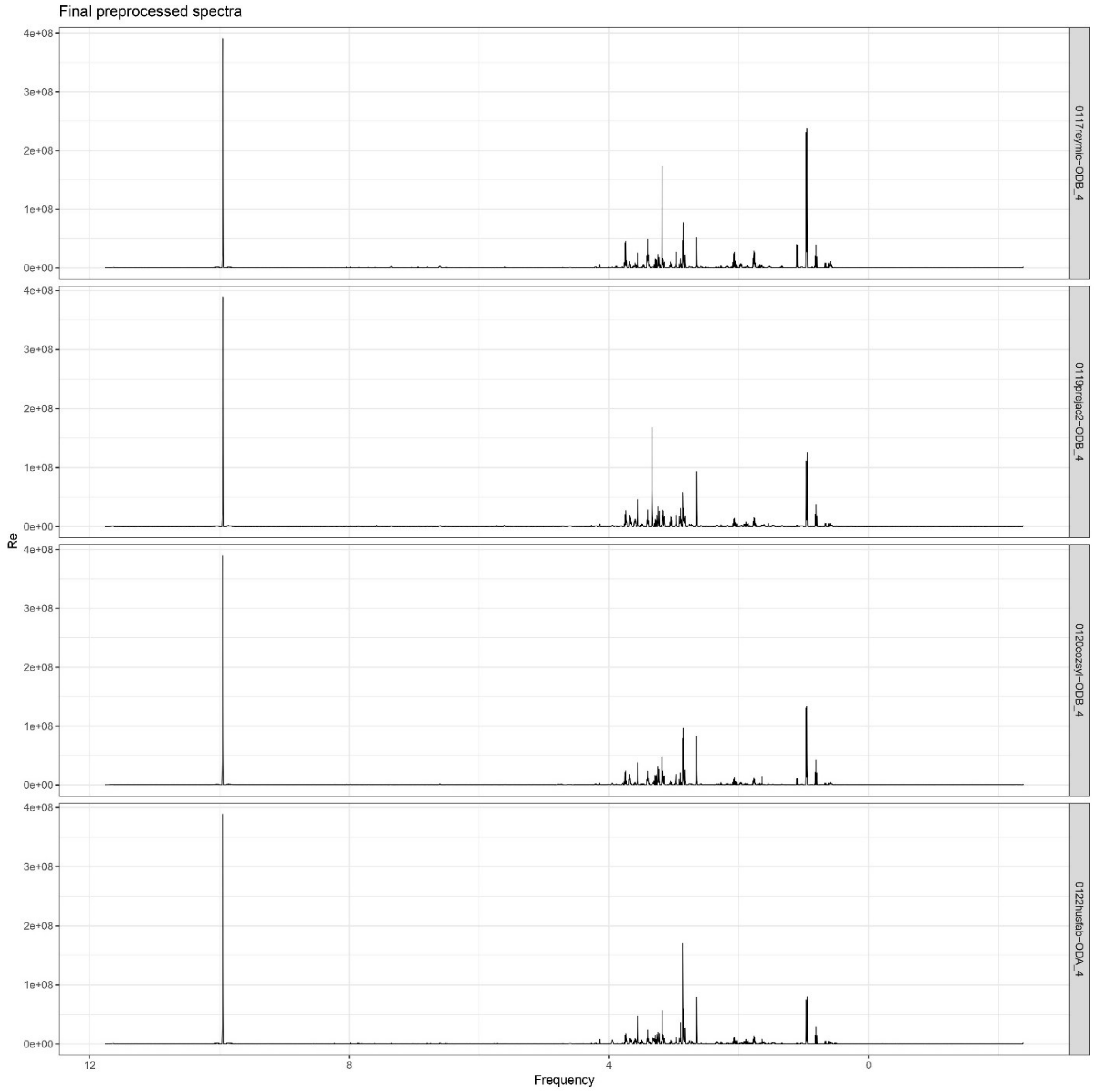

Figure S3 (n). Individual spectra (n = 71) from MetaboLights study MTBLS869 after being read and preprocessed through Workflows4Metabolomics. Samples 0117reymic-ODB_4, 0119prejac2-ODB_4, 0120cozsyl-ODB_4, and 0122husfab-ODA_4.

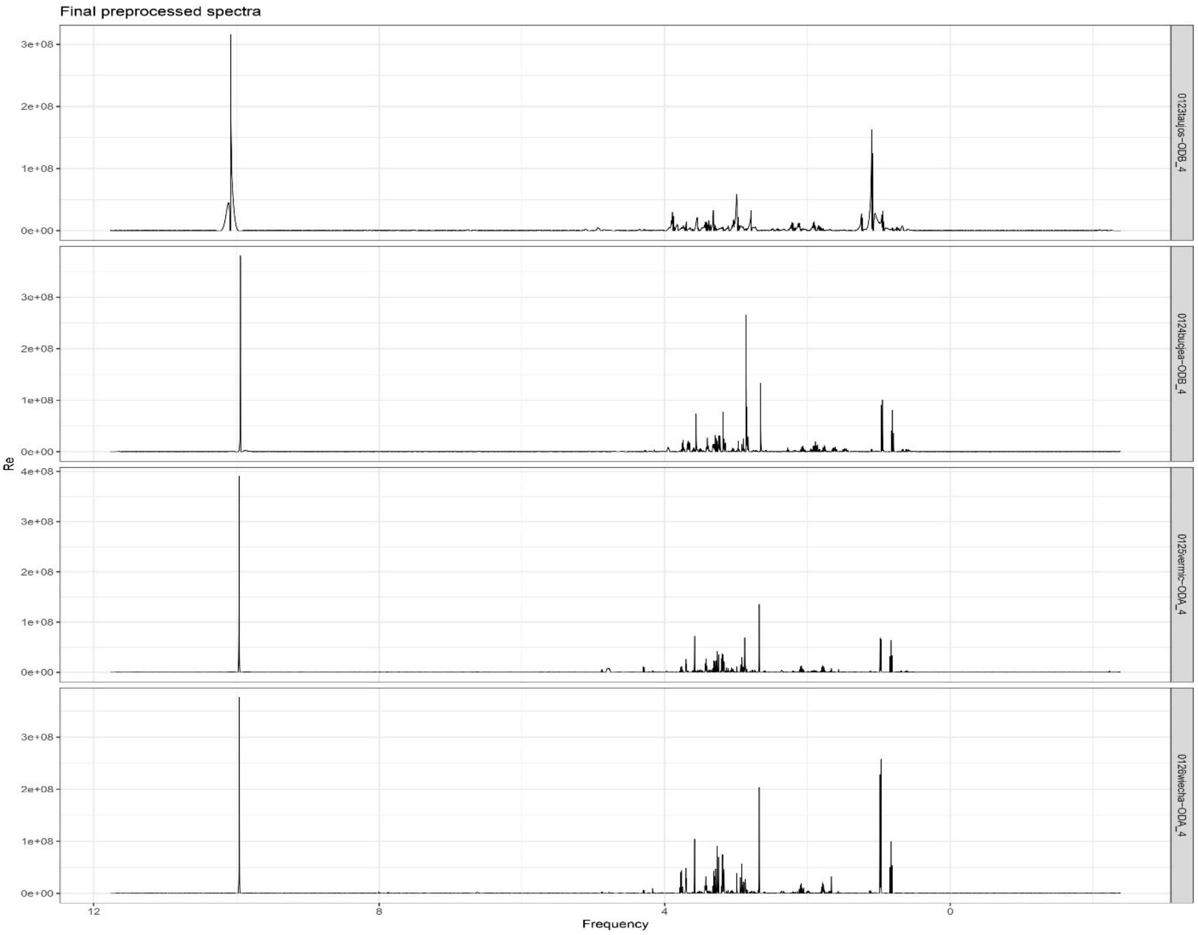

Figure S3 (o). Individual spectra (n = 71) from MetaboLights study MTBLS869 after being read and preprocessed through Workflows4Metabolomics. Samples 0123taujos-ODB_4, 0124bucjea-ODB_4, 0125vermic-ODA_4, and 0126wiecha-ODA_4.

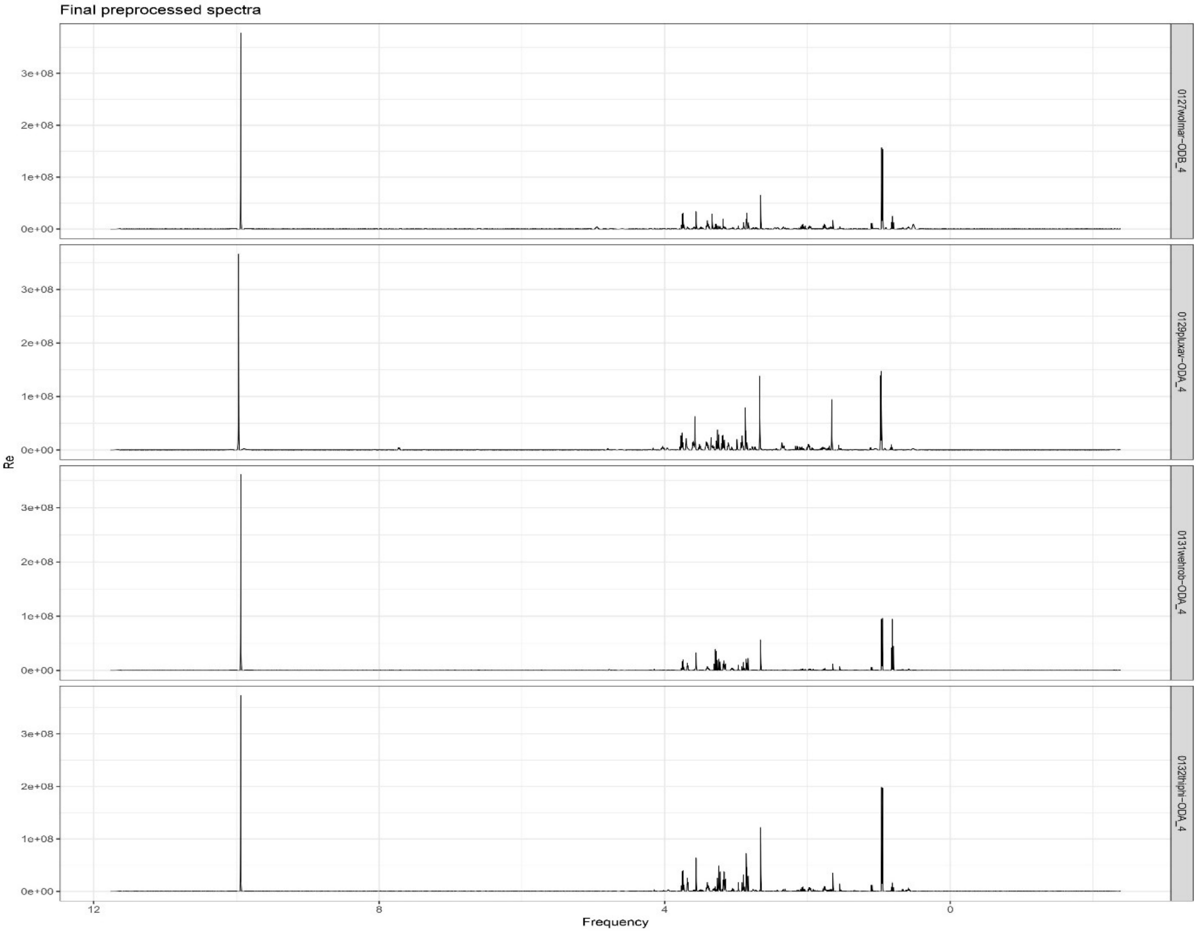

Figure S3 (p). Individual spectra (n = 71) from MetaboLights study MTBLS869 after being read and preprocessed through Workflows4Metabolomics. Samples 0127wolmar-ODB_4, 0129pluxav-ODA_4, 0131wehrob-ODA_4, and 0132thiphi-ODA_4.

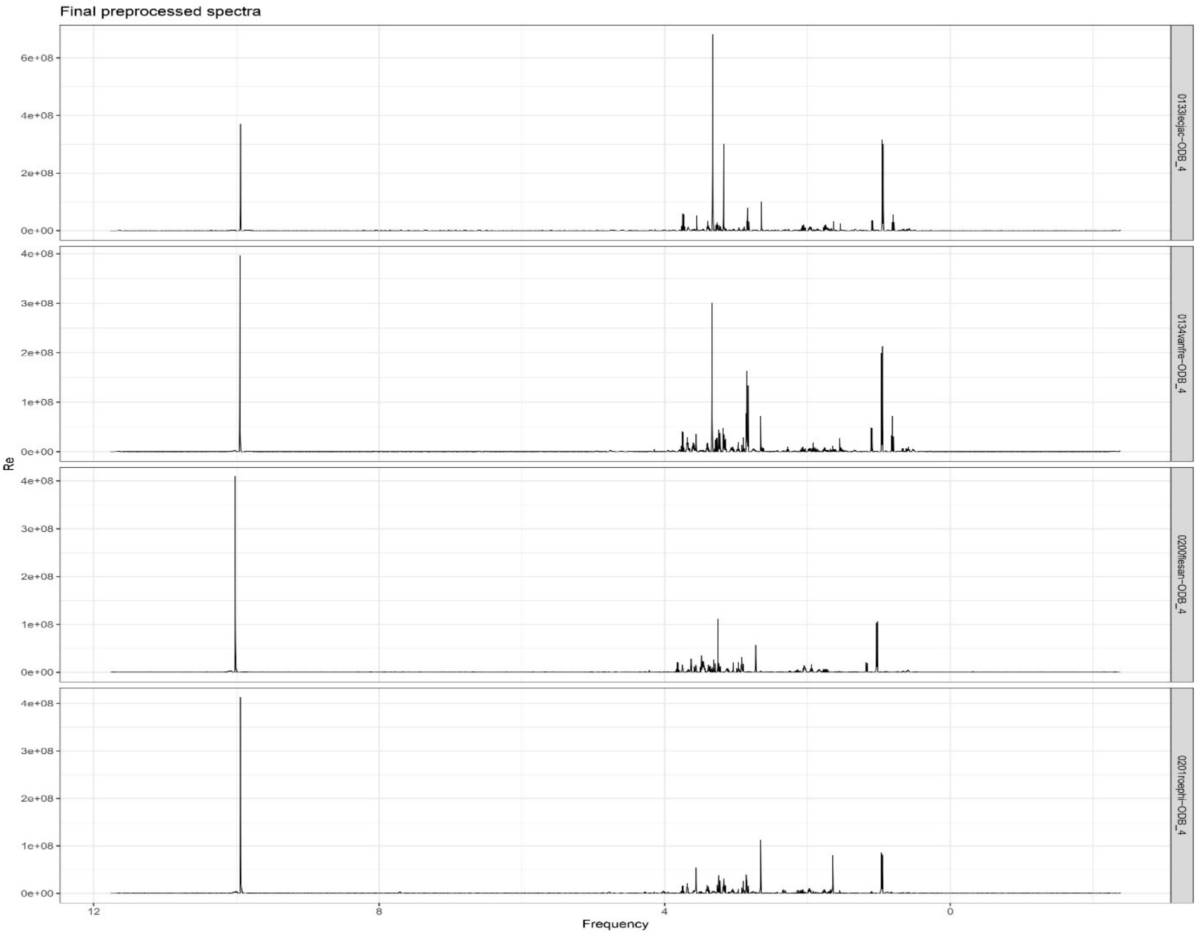

Figure S3 (q). Individual spectra (n = 71) from MetaboLights study MTBLS869 after being read and preprocessed through Workflows4Metabolomics. Samples 0133lecjac-ODB_4, 0134wanfre-ODB_4, 0200flesan-ODB_4, and 0201roephi-ODB_4.

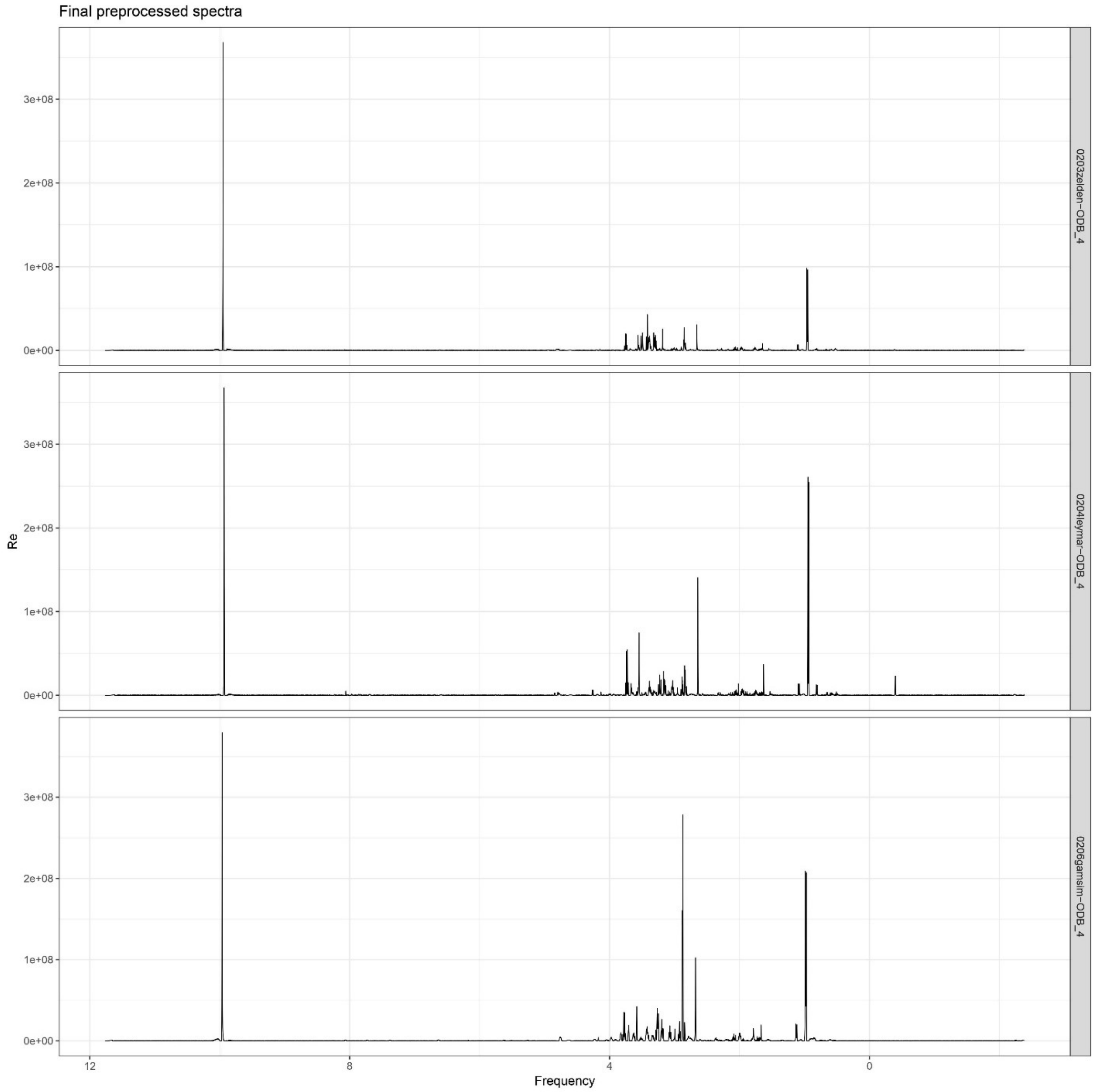

Figure S3 (r). Individual spectra (n = 71) from MetaboLights study MTBLS869 after being read and preprocessed through Workflows4Metabolomics. Samples 0203zelden-ODB_4, 0204leymar-ODB_4, and 0206gamsim-ODB_4.

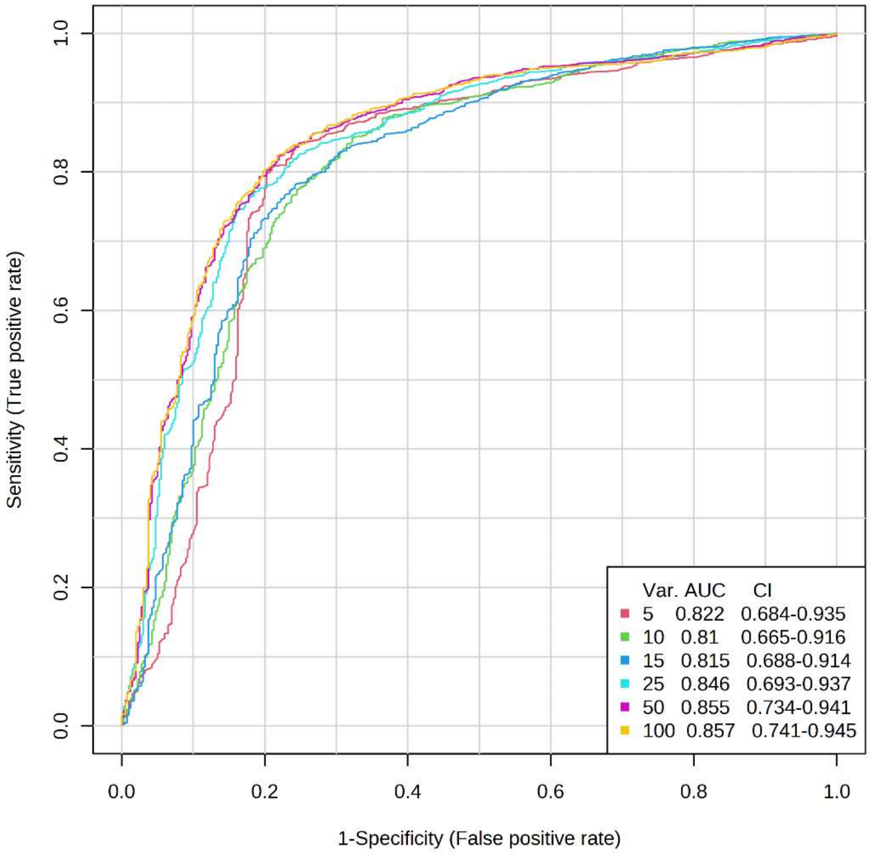

Figure S4. ROC curves for the discrimination of control and bacterial samples from the MetaboLights study with id MTBLS563. ROC curve analysis was generated by Monte-Carlo cross-validation using balanced sub-sampling and performed based on PLS-DA. Of note, control sample 1 and bacterial sample 26 were considered outliers based on an exploratory sPLS-DA model and therefore were discarded prior to the classification analysis. Also, samples were normalized by median, and features were log-transformed and mean-centered.

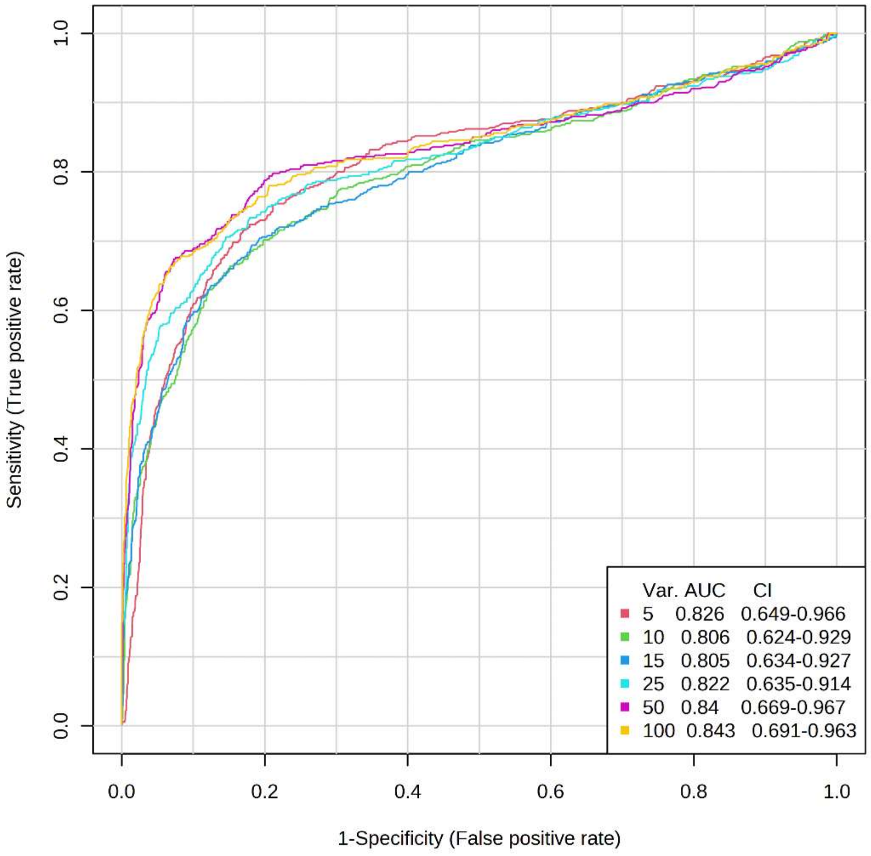

Figure S5. ROC curves for the discrimination of control and viral samples from the MetaboLights study with id MTBLS563. ROC curve analysis was generated by Monte-Carlo cross-validation using balanced sub-sampling and performed based on PLS-DA. Of note, control samples 1 and 55 were considered outliers based on an exploratory sPLS-DA model and therefore were discarded prior to the classification analysis. Also, samples were normalized by median, and features were log-transformed and mean-centered.
